# A near-infrared light-controlled smart nanocarrier with reversible polypeptide-engineered valve for targeted fluorescence-photoacoustic bimodal imaging-guided chemo-photothermal therapy

**DOI:** 10.7150/thno.37047

**Published:** 2019-10-14

**Authors:** Cheng Li, Xiao-Quan Yang, Jie An, Kai Cheng, Xiao-Lin Hou, Xiao-Shuai Zhang, Xian-Lin Song, Kai-Chen Huang, Wei Chen, Bo Liu, Yuan-Di Zhao, Tian-Cai Liu

**Affiliations:** 1 Britton Chance Center for Biomedical Photonics at Wuhan National Laboratory for Optoelectronics-Hubei Bioinformatics & Molecular Imaging Key Laboratory, Department of Biomedical Engineering, College of Life Science and Technology, Huazhong University of Science and Technology, Wuhan 430074, Hubei, P. R. China.; 2 Beijing Advanced Innovation Center for Big Data-Based Precision Medicine, School of medicine, Beihang University, Beijing 100191, P. R. China.; 3 Key Laboratory of Biomedical Photonics (HUST), Ministry of Education, Huazhong University of Science and Technology, Wuhan 430074, Hubei, P. R. China.; 4 Institute of Antibody Engineering, School of Laboratory Medicine and Biotechnology, Southern Medical University, Guangzhou 510515, Guangdong, P. R. China.; 5 Guangdong Provincial Key Laboratory of Construction and Detection in Tissue Engineering, Southern Medical University, Guangzhou 510515, Guangdong, P. R. China.

**Keywords:** cancer therapy, charge reversible, drug delivery, protein engineering, dendritic mesoporous silica

## Abstract

Despite burgeoning development of nanoplatform made in the past few years, it remains a challenge to produce drug nanocarrier that enables requested on/off drug release. Thus, this study aimed to develop an ideal near-infrared light-triggered smart nanocarrier for targeted imaging-guided treatment of cancer that tactfully integrated photothermal therapy with chemotherapy to accurately control drug release time and dosage.

**Methods:** This delivery system was composed of Ag_2_S QD coating with dendritic mesoporous silica (DMSN), which acted as nanocarrier of doxorubicin localized inside pores. To provide the nanocarrier with controlled release capability, a polypeptide-engineered that structure was reversible to photothermal effect of Ag_2_S QD, was covalently grafted to the external surface of drug-loaded DMSN.

**Results:** This nanocarrier with the size of 40~60 nm had satisfactory biocompatibility and photothermal conversion efficiency up to 28.35%. Due to acidity-triggered charge reversal of polypeptide, which significantly extended circulation time and improved targeting ability, fluorescence and photoacoustic signals were still obvious at tumor site post-24 h by tail vein injection and chemo-photothermal synergistic therapy obviously enhanced antitumor efficacy. Mild PTT with multiple short-term exposures not only reduced the side effect of overdose drug but also avoided skin damage caused by long-term irradiation.

**Conclusion:** By adjusting irradiation time and on/off cycle, multiple small amount local drug release reduced the side effect of overdose drug and skin damage. This novel approach provided an ideal near-infrared light-triggered nanocarrier with accurate control of area, time, and especially dosage.

## Introduction

Photothermal therapy (PTT) has attracted widespread interest owing to its invasiveness and specific spatial and temporal selectivity. Many materials act as efficient photothermal converter, such as metal nanomaterials (gold nanorod 1 and gold nanocage 2), semiconductor quantum dots (*e.g.*, CuS 3 and Bi_2_S_3_ 4), carbon nanomaterials (carbon dot 5 and carbon nanotube 6) and some two-dimensional nanostructures (graphene 7 and MoS_2_ nanosheet 8 ). However, PTT is insufficient to eradicate tumor cell at once that tumor is easy to relapse due to the uneven heat distribution when it is used alone. Chemotherapy, the traditional therapy for clinical treatment, due to the non-specific distribution of drug has already caused serious problems such as side effect and drug resistance, the drawbacks of single treatment are becoming increasingly apparent. Since PTT could increase cell membrane permeability to further improve the uptake rate of drug and enhance the toxicity of chemotherapy 6,9-11, while the release of drug can also offset the defect of easy recurrence of tumor in the later stage of PTT. Therefore, PTT combined with other therapy has become a promising method for efficient tumor ablation and minimally invasive treatment 12-14. One approach is to use a stimulus-responsive targeting drug-carrier, which combines PTT together with on-demand release of local high-concentration drug to cover a large treatment area helping to achieve success in killing the whole cancer cell population 15-17. The latest research reported that Ag_2_S QD not only was an fluorescence imaging agent, but also had ideal photoacoustic effect and high photothermal conversion efficiency 18,19, indicating that it was an excellent multifunctional nanomaterial for PTT as well as bimodal imaging of tumor* in vivo*.

Dendritic mesoporous silica (DMSN) with unique central-radial structure is emerging as a promising nanocarrier for biomedical application owning to large pore size and highly surface area compared with conventional mesoporous silica nanoparticle 20-22. Whereas leakage of drug during the transportation of drug delivery system is a problem that has to face in the application *in vivo*. Stimulus-responsive drug controlled release system, an intelligent delivery system, which can respond to various external or internal stimuli, such as pH 23, temperature 2, enzyme 24, redox agent 25, magnetic field 26 and light 7. A promising drug release mechanism is through the engagement of NIR light, which switchable controls drug on/off release from a photothermal nanocarrier through the change of thermosensitive material at a desired time 27,28. Such a spatial-temporal control in drug management may significantly improve the drug efficacy in treatment. At present, thermosensitive materials have been widely used in NIR light stimulation response drug delivery system 29,30. However, these materials still have defects in tumor targeting, biocompatibility, surface modifiability and reversible valve, so it is urgent to find new temperature sensitive materials to overcome these shortcomings. Genetically engineered polypeptide was a kind of polypeptide that could be easily and flexibly designed into precise structure by DNA recombinant technology at molecular level. The DNA sequence of interest is modified as required to express amino acid sequence with specific functions, endowing polypeptide with targeting property (*e.g.*, RGD), modifiable group (*e.g.*, mercapto cysteine), or temperature-sensitive reversible dynamic self-assembly structure 31-33. In addition, the excellent biodegradability and biocompatibility of polypeptide also bring broad prospects for their application *in vivo*. Therefore, it is a better choice to use DNA coding technology to prepare thermosensitive genetically engineered polypeptide for NIR light triggered on-demand drug delivery system.

**Figure 1:**
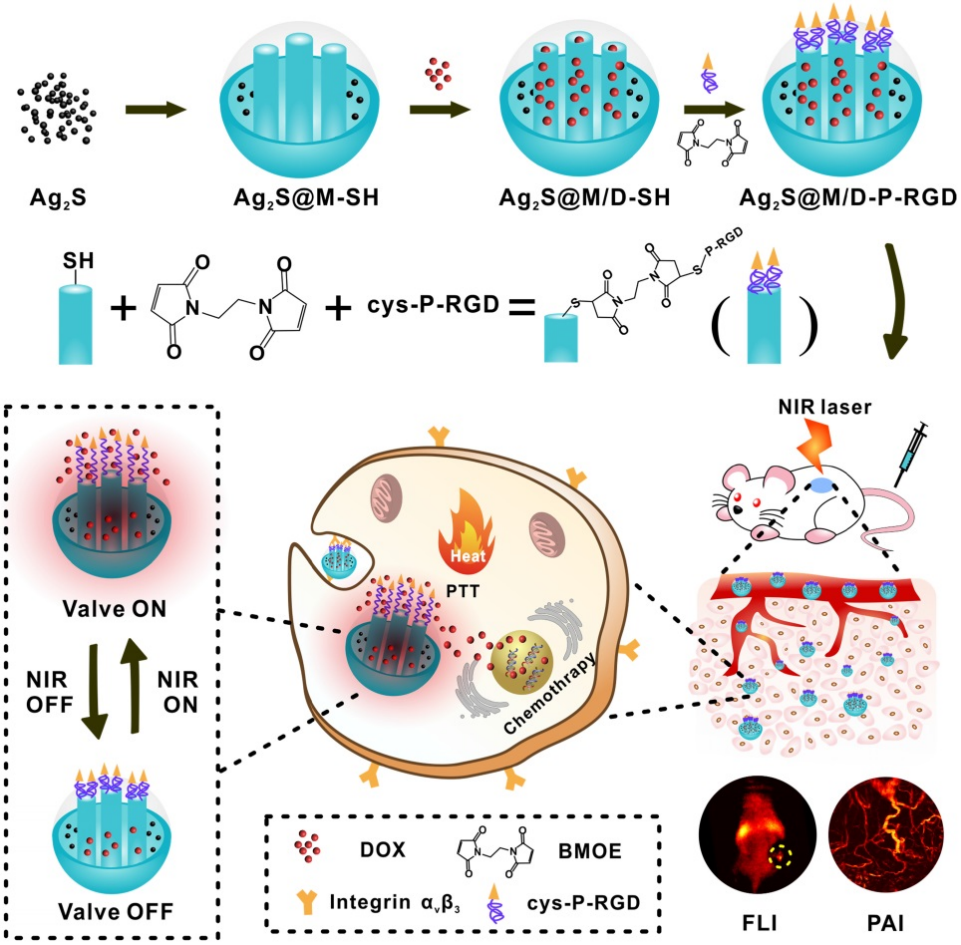
Schematic illustration of the synthesis route of Ag_2_S@M/D-P-RGD, tumor targeted fluorescence-photoacoustic imaging and combined chemo-photothermal therapy.

Hence, we designed a novel NIR light-triggered multifunctional nanocarrier with tumor targeted fluorescence-photoacoustic bimodal imaging and chemo-photothermal combined therapy (Figure 1). Firstly, doxorubicin (DOX) was loaded into DMSN coated Ag_2_S QD delivery system prepared by one-pot method. Followed, genetically engineering polypeptide cys-P-RGD containing cysteine and RGD sequence was covalently grafted to the external surface of DMSN by crosslinking agent to block drug premature leakage and generated Ag_2_S@M/D-P-RGD. The distribution of Ag_2_S@M/D-P-RGD was monitored by fluorescence imaging (FLI) and photoacoustic imaging (PAI) *in vivo*, and after the targeting probe was preferentially taken up by tumor cell through receptor-mediated endocytosis, NIR irradiation not only caused PTT but also triggered polypeptide unfolding to release drug. Without NIR irradiation, the polypeptide recovered to pentamer to block the pore and stopped drug release. The release amount of drug could be on-demand controlled by adjusting the irradiation time and laser on/off cycle. More importantly, this multifunctional nanocarrier had excellent biocompatibility and tumor targeting ability, and mild PTT with short-term and multiple cycle laser irradiation could significantly improve the efficacy while reducing side effect and skin damage. This robust nanoplatform for tumor targeted imaging and on-command controlled combinational therapy might provide an insight for cancer theranostics.

## Results and Discussion

### Synthesis and characterization of Ag_2_S@M-P-RGD

**Figure 2:**
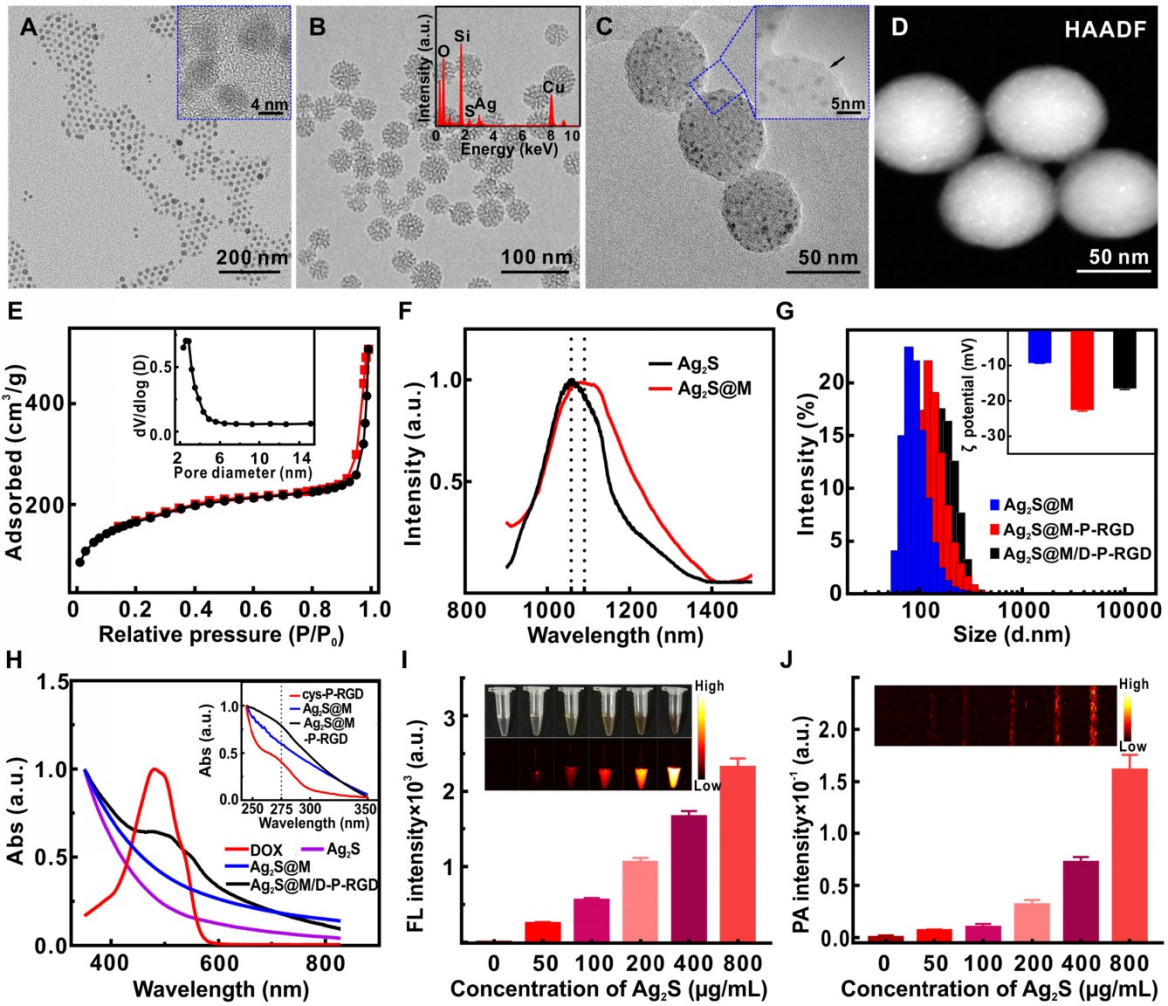
TEM and high-resolution TEM (inset) images of Ag_2_S QD (A), Ag_2_S@M (B) and EDX spectrum (inset), and Ag_2_S@M-P-RGD (C); high-angle annular dark field image (HAADF) of Ag_2_S@M (D); nitrogen adsorption-desorption isothermal curve (E) and pore size distribution (inset); fluorescence spectra of Ag_2_S QD and Ag_2_S@M (F); hydrated particle size distribution (G), zeta potentials (inset) and UV-Vis spectra (H) of different nanoparticles in pure water; fluorescence (I) and photoacoustic (J) images of Ag_2_S@M at different concentrations.

Based on Stöber mechanism and sol-gel chemical reaction, 5.2 nm oil-soluble Ag_2_S QD with good crystal structure and prominent lattice (Figure 2A) was embedded in DMSN by anion assisted approach to obtain uniform spherical Ag_2_S@M nanoparticle. DMSN with the unique topology of central-radial dendritic structure was prepared with CTAB and NaSal as structure-directing agents, TEOS as silicon source and TEA as catalyst. According to the existing literature 34, we speculated that the pore enlarging mechanism was presumably due to the presence of Sal^-^. It was proposed that the interaction between Sal^-^ and CTA^+^ micelle was energetically favored due to their high miscibility and strong electrostatic attraction, and eventually Sal^-^ migrated into the micelle with its hydrophobic part embedded in the hydrophobic region of micelle. The easy penetration of Sal^-^ with large hydrophobic portion into the micelle leaded to the increase in packing parameter, which induced the micelle structural transition toward vesicular/lamellar structure and eventually formed the central radial structure as monitored by time dependent study for Ag_2_S@M. To further understand the effect of the molar ratio (*φ*) of NaSal/CTAB on formation of dendritic structure of Ag_2_S@M, systematic studies had been conducted (Figure S1). When *φ* was increased from 0:1 to 0.25:1, the particle size increased from 40 nm to 80 nm; further increasing *φ* to 0.5:1 and 0.75:1 leaded to significantly rise of Ag_2_S@M particle size and pore diameter, and the structure of central-radial channel was becoming clearer. When *φ* was 1:1, the edge of the particle began to appear wrinkles and incomplete. Considering that nanoparticle around 50 nm was ideal for nanocarrier *in vivo* 35, 36, *φ* was finally chosen as 0.1:1 and the size of Ag_2_S@M was about 40~60 nm (Figure 2B). HAADF result showed that Ag_2_S QD was uniformly distributed in DMSN (Figure 2D) and EDX spectrum (Figure 2B, inset) confirmed that the atomic ratio of Si, Ag and S was 82.8:12.3:5.0, indicating that Ag_2_S QD had been encapsulated in DMSN, and the content of Ag_2_S QD in Ag_2_S@M was calculated to be 6.73 % (w/w) by graphite furnace atomic absorption method. N_2_ adsorption/desorption isotherm curve (Figure 2E) presented typical IV type isotherm, uniform mesopores generated capillary condensation step at P/P_0_=0.2~0.5 and the H1 hysteresis loop at high pressure region P/P_0_=0.8~0.9 reflected the disordered mesoporous structure. BET surface area was 455.19 m^2^/g, total pore volume was 0.47 cm^3^/g, and pore size calculated by BJH method was 3.84 nm (Figure 2E, inset).

Fluorescence spectrum showed that the maximum emission of oil-soluble Ag_2_S QD was about 1060 nm in NIR-II region under 808 nm excitation, emission peak emerged a slight red shift after DMSN coating and the fluorescence quantum yield was about 3.6±0.71 % (Figure 2F). Ag_2_S@M presented strong fluorescence and photoacoustic signals (Figure 2I and J), both intensity enhanced gradually with Ag_2_S concentration increased, laying a good foundation for *in vivo* multi-mode imaging. The photothermal effect of Ag_2_S@M was concentration dependence (Figure S2A and C) and laser power intensity dependence (Figure S2B and D). After five laser irradiation cycles, there was no obvious decline trend of photothermal conversion (Figure S2E), and TEM results revealed that silicon shell skeleton did not scatter, and still maintained central-radial dendritic structure (Figure S3), indicating that it had good photothermal stability. The photothermal conversion efficiency (*η*) of Ag_2_S@M was calculated to be 28.35 % (Figure S2F). The imaging characteristics and photothermal conversion efficiency of Ag_2_S QD coated by DMSN were consistent with that of Ag_2_S QD (Figure S4 and S5). Therefore, Ag_2_S@M had an outstanding photostability and photothermal effect for PTT.

The negatively charged polypeptide cys-P-RGD or cys-P was covalently bonded to mercapto-modified Ag_2_S@M surface *via* maleimide at the end of the crosslinking agent BMOE. The hydrodynamic size of Ag_2_S@M-P-RGD increased to 131.6±3.12 nm compared with Ag_2_S@M and zeta potential also rose to -22.4±0.52 mV (Figure 2G); UV-vis absorbance spectra showed characteristic absorption peak of polypeptide at 275 nm (Figure 2H, inset) and the HTEM also demonstrated that the pore was covered with a thin layer of substance (Figure 2C, inset). FITC modified cys-P-RGD was also bonded to Ag_2_S@M surface (Ag_2_S@M-P-RGD/FITC) and thoroughly cleaned to determine fluorescence (Figure S6). Fluorescence spectrum showed that the probe had FITC fluorescence characteristic peak, and the solution showed obvious green fluorescence compared with the unmodified group. The above results indicated that the polypeptide cys-P-RGD was successfully coated on the surface of Ag_2_S@M. As positively charged drug DOX was adsorbed in the pore, the zeta potential of Ag_2_S@M/D-P-RGD decreased relatively (-16.27±0.55 mV) and characteristic absorption peak of DOX appeared at 490 nm (Figure 2H), which proved that DOX had been loaded into the pore. Particle stability test illustrated that temperature (in the range of 4~37 ºC) and storage environment had little effect on Ag_2_S@M-P-RGD (Figure S7).

### Acidity-triggered charge reversion and excellent biocompatibility depend on polypeptide

**Figure 3:**
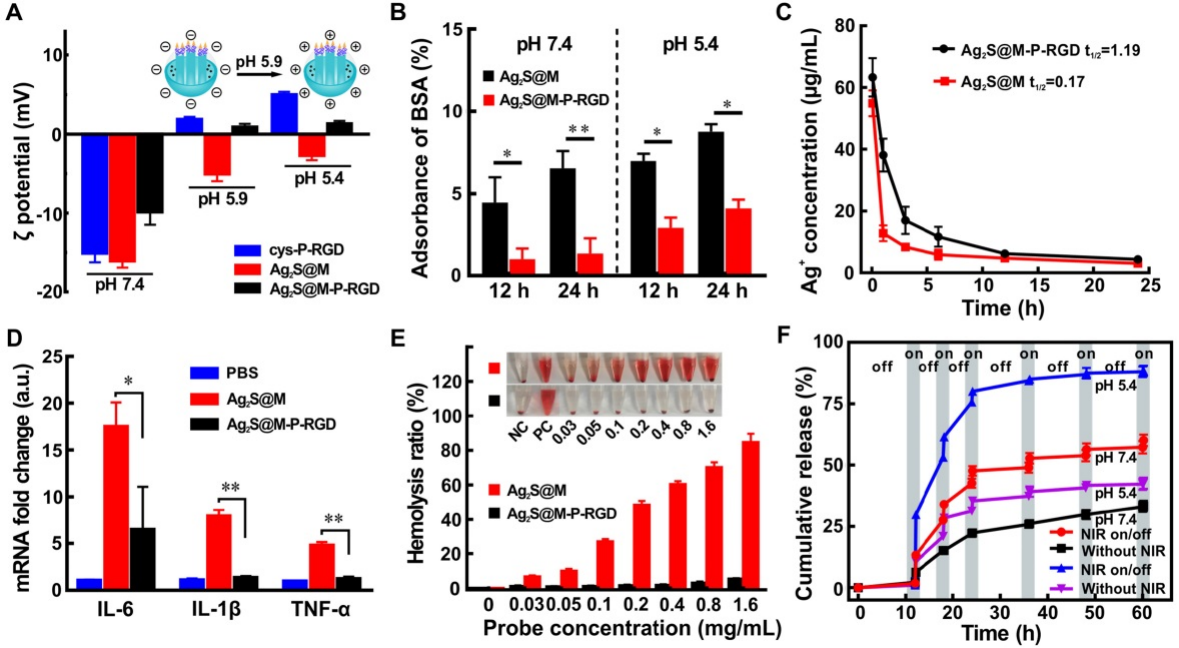
Zeta potential changes of cys-P-RGD, Ag_2_S@M and Ag_2_S@M-P-RGD under different pH conditions (A); relative protein adsorptions of Ag_2_S@M and Ag_2_S@M-P-RGD (0.4 mg/mL) incubated with BSA for 12 h and 24 h under different pH conditions (B); pharmacokinetics of Ag_2_S@M and Ag_2_S@M-P-RGD (100 mg/kg) (C); mRNA expression levels of the inflammatory cytokines, IL-6, IL-1β and TNF-α, were assessed in RAW 264.7 macrophages incubated with Ag_2_S@M and Ag_2_S@M-P-RGD (100 µg/mL) for 24 h (D); hemolysis of Ag_2_S@M and Ag_2_S@M-P-RGD (E); DOX release curves of Ag_2_S@M/D-P-RGD (2.5 mg/mL) with and without laser irradiation at pH 7.4 and 5.4 (F). *: *p*<0.05, **: *p*<0.01.

Various acidic and basic functional groups on amino acid side chain and termini of protein and polypeptide may be charged, making them biological amphoteric ionization property. Amino acids that make up protein may be positive, negative, neutral, or polar in nature, and together give a protein its overall charge. The isoelectric point (pI) of a peptide is defined as the pH at which the peptide has a net charge of zero. At pH below the pI, peptide presents a net positive charge; above the pI, it presents a net negative charge. The isoelectric point (pI) of polypeptide cys-P-RGD designed by us was calculated to be 6.26, which theoretically would be negatively and positively charged in neutral blood and acidic tumor tissue microenvironment, respectively. Since the pI of BSA is 4.7 37, the acidic environment of tumor could not cause its charge reversion, it was used as control to an analyze the acidity-triggered charge reversion of cys-P-RGD (Figure S8). The results showed that with the increase of environmental acidity (pH 6.2→5.8), the charge of polypeptide-modified probe Ag_2_S@M-P-RGD began to change from negative to positive, while BSA-modified probe remained negative due to the environmental pH was above its pI. When Ag_2_S@M-P-RGD was incubated in PBS (0.01 M) at pH 5.9 for 2 h, zeta potential transformed to +0.89±0.40 mV compared with -9.85±1.60 mV under neutral condition (Figure 3A). In a more acidic environment (pH 5.4), the zeta potential of polypeptide cys-P-RGD reversed to +4.97±0.38 mV, and the surface charge of Ag_2_S@M-P-RGD also reversed to +1.31±0.35 mV, while Ag_2_S@M still maintained electronegative. Hence, negatively charged polypeptide-modified probe would also reject the adsorption of negatively charged proteins in blood during blood circulation, avoiding the formation of protein corona to be captured by immune organs, thus extending the blood circulation time and improving the enhanced permeability and retention (EPR) effect 38-40. After the probe arrived at tumor site, as the extracellular pH in the tumor microenvironment was generally around 6, the surface polypeptide would be positive charge, which was more conducive to anchoring on the surface of negatively charged cell membrane, and further enriched in the tumor cells. Once the probe was endocytosis by lysosome, the acidity was further increased, and the positive charge on the probe surface was more conducive to its escape.

Moreover, BSA was used to investigate the interaction of Ag_2_S@M-P-RGD with the abundant plasma proteins in blood, which was one major serum protein often found in the protein corona of nanoparticle. After incubation for 12 and 24 h under neutral condition (pH 7.4), significantly less BSA was found on Ag_2_S@M-P-RGD compared with Ag_2_S@M, indicating that negatively charged polypeptide on surface prevented proteins from adsorbing on the surface, whereas this prevention was not happened on Ag_2_S@M. However, the adsorption of BSA by Ag_2_S@M-P-RGD increased observably under acidic condition (pH 5.4), which proved that nanoprobe reversed to positive charge were beneficial to anchor negatively charged cell membrane proteins (Figure 3B). Pharmacokinetic results also showed that the circulation half-life of Ag_2_S@M-P-RGD was calculated to be 1.19 h (Figure 3C), which was obviously better than 0.17 h of Ag_2_S@M. Therefore, this charge reversal helped to avoid the formation of protein crown in blood circulation, prolong the circulation time and increase the enrichment of Ag_2_S@M-P-RGD in tumor site.

When nanoparticle entered the host, it interacted with macrophage to trigger immune response and caused inflammatory effect. IL-6, IL-1β and TNF-α, as inflammatory mediators, are typically used as markers for judging acute macrophage-related inflammatory reaction 41. The results showed that the mRNA levels of IL-6, IL-1β and TNF-α in RAW 264.7 macrophages incubated with Ag_2_S@M-P-RGD were significantly lower than those of cells incubated with Ag_2_S@M (Figure 3D), indicating that the modification of polypeptide cys-P-RGD significantly reduced the inflammatory response to nanoprobe (*p*<0.01). In addition, the hemolysis of nanoparticle would severely limit its delivery* in vivo*, then such experiment was also carried out for nanoprobe before and after polypeptide modification, the results showed that Ag_2_S@M-P-RGD did not trigger hemolysis at concentration up to 1.6 mg/mL (Figure 3E), while the hemolytic behavior of Ag_2_S@M that damaged lipid bilayer occurred at a lower concentration. Therefore, the modification of polypeptide improved the biocompatibility of inorganic silicon sphere, and the modified nanoprobe intravenous administration was non-toxic to red blood cells, providing feasibility for *in vivo* tumor treatment.

### NIR-triggered on-demand drug release of Ag_2_S@M/D-P-RGD

P domain was the coiled-coil region of cartilage oligomeric matrix protein, which could self-assemble into pentamer at room temperature and was sensitive to temperature change 42,43. Once the temperature rose, the hydrogen bond between peptides became break, resulting in the unfolding and looseness of polypeptide structure, while the pentamer structure could be resumed with temperature cooling. A RGD sequence, specifically binding to the integrin α_v_β_3_ expressed by cancer cells, was added at N-terminal of P domain by DNA recombinant technology to improve the tumor targeting ability, and a mercapto cysteine was introduced to C-terminal for further modification. Circular dichroism was used to study the thermosensitive characteristic of cys-P-RGD. The spectra showed that the *α*-helix signal of polypeptide at 208 and 222 nm gradually decreased with the increase of temperature (Figure S9A), indicating that the pentamer structure gradually loosed. However, the *α*-helix signal gradually re-increased to original state with the re-decrease of temperature (Figure S9B), indicating that the structure of cys-P-RGD was also temperature sensitive. The thermosensitive cys-P-RGD was used as a “gatekeeper” which endowed Ag_2_S@M/D-P-RGD with the ability to accurately control drug on-demand release through reversible change of polypeptide structure. Here, DOX was adsorbed into pore, the loading rate and encapsulation efficiency reached up to 5.31±0.42 % and 58.42±4.65 %, respectively. After 12 h, only a little DOX leaked out without laser radiation (Figure 3F). However, the drug release of Ag_2_S@M/D-P-RGD increased rapidly with the temperature rise caused by laser irradiation, and with the “on-off-on” of laser irradiation, drug release also followed “burst-zero-burst” staircase-shaped profile, suggesting the thermal-induced release was an on-demand stoppable process. Due to the acidic environment of tumor, the drug release of Ag_2_S@M/D-P-RGD at pH 5.4 was evaluated same as the previous experiment and the cumulative releases of DOX was 88.43±2.14 % under “on-off-on” laser irradiation, higher than 60.11±2.32 % at pH 7.4. Therefore, the acidic environment at the tumor site was more conducive to drug release. In order to verify the contribution of thermo-responsiveness of polypeptide cys-P-RGD in drug release, the release curves of Ag_2_S@M/D and Ag_2_S@M/D-BSA with and without laser irradiation were respectively conducted at pH 7.4 and 5.4 (Figure S10). The results showed that the release curve of Ag_2_S@M/D without laser irradiation presented uncontrolled fast drug release rate at pH 7.4; after 60 h, 53.97±0.57 % DOX was released due to the lack of blocking compared with Ag_2_S@M/D-P-RGD group (33.33±2.12 % at pH 7.4). And the high temperature induced by NIR naturally increased the diffusion rate of encapsulated DOX, resulting in high uncontrolled fast release (77.87±0.97 % after 60 h at pH 7.4). However, for Ag_2_S@M/D-P-RGD (Figure 3F), the DOX release was slower before and after laser irradiation (60.11±2.32 % after 60 h at pH 7.4). proving that cys-P-RGD played an active role in thermo-responsiveness release. In addition, as the thermal denaturation temperature of BSA is about 65 °C compared with 48 °C of cys-P-RGD, it was relatively stable in the laser controlled release process, thus serving as a control for analyzing the NIR-governed drug release of cys-P-RGD (Figure S10B). The results showed that the total release amount was relatively lower than that of cys-P-RGD-modified probe after laser irradiation, and excessive thermal denaturation temperature was not conducive to the full release of drug. The drug release also occurred in without NIR irradiation groups or “off” status. We speculate that when the small molecule drug DOX was blocked into DMSN, some drug would loaded into the peptide pentamer, resulting in a small amount of leakage without NIR laser irradiation and “off” status. In addition, an increase in temperature would have a negative impact on protein structures, and its initial state could not be completely restored, resulting in the drug leakage. All in all, the cys-P-RGD modified Ag_2_S@M/D-P-RGD could prevent drug leakage, moreover, it could accurately control drug release through laser on/off and irradiation time. Meanwhile, the increase in temperature increased the permeability of nanoparticle, drug diffusion rate and oxygen content (heat increased blood circulation rate), and further improved the therapeutic effect 44, 45.

### Cytotoxicity and targeting ability of Ag_2_S@M/D-P-RGD

**Figure 4:**
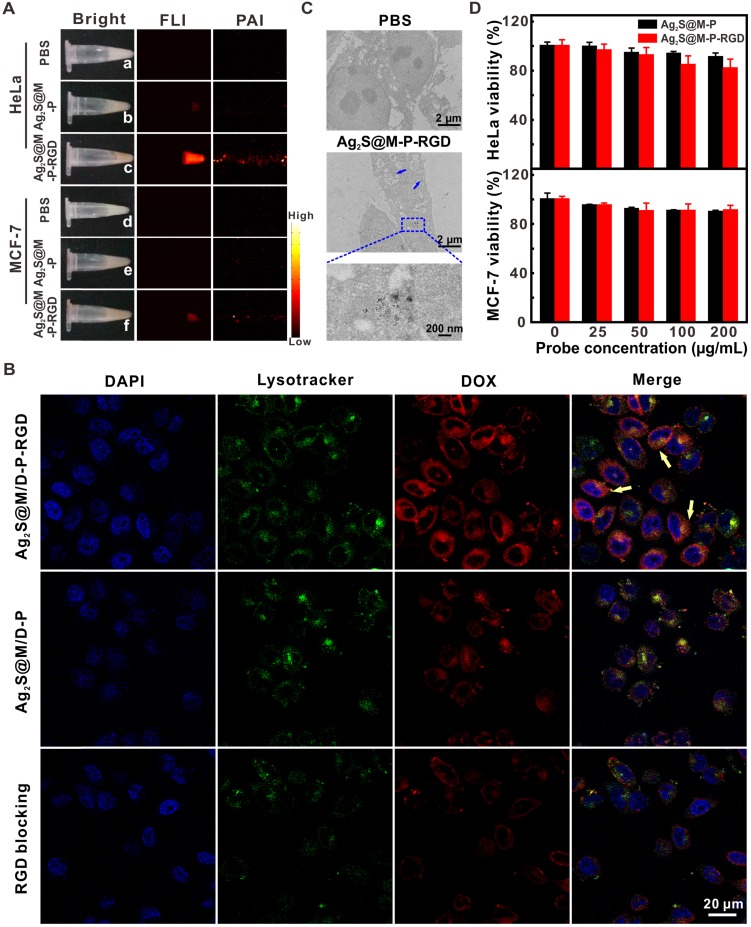
White light, NIR fluorescence and photoacoustic images of HeLa and MCF-7 cells incubated with Ag_2_S@M/D-P and Ag_2_S@M/D-P-RGD (A); CLSM images of HeLa cells incubated with Ag_2_S@M/D-P, Ag_2_S@M/D-P-RGD and Ag_2_S@M/D-P-RGD with c(RGDyK) pre-blocking (denoted as “RGD blocking”) (B); biological TEM images of Ag_2_S@M-P-RGD incubated with HeLa cells (C); survival rate of HeLa and MCF-7 cells incubated with Ag_2_S@M-P and Ag_2_S@M-P-RGD at different concentrations (D).

To test targeting specificity of probe, HeLa and MCF-7 cells with high and low expression of integrin α_v_β_3_ were used as positive and negative cells, respectively. After the probe that with or without RGD modification was incubated with both cells for 4 h, NIR fluorescence and photoacoustic images revealed that HeLa cells incubated with Ag_2_S@M-P-RGD exhibited the strongest signal (Figure 4A), and little non-specific adsorbed probe also caused weak signal in other groups. By incubating HeLa cells with both types of probes for 4 h, confocal images showed that RGD-modified Ag_2_S@M/D-P-RGD transported the most DOX into HeLa cells, and red fluorescence signal of DOX was mainly distributed in cytoplasm and co-localized with the lysotracker (Figure 4B). TEM at cell level also showed similar result (Figure 4C). In contrast, the fluorescence of cells in either the negative probe group or free RGD pre-blocked positive probe group was weaker, indicating that the uptake rates of probes reduced significantly. Therefore, cells engulfed RGD-modified probe *via* the receptor-mediated endocytosis pathway.

Biocompatibility of probe should be the first consideration for following bio-application. The toxicities of probes without DOX (Ag_2_S@M-P-RGD and Ag_2_S@M-P) were investigated for normal cell (3T3 cells) and cancer cells (HeLa and MCF-7 cells) by a standard assay. After incubation of 3T3 cells with 200 μg/mL of Ag_2_S@M-P-RGD and Ag_2_S@M-P, the cells of the two groups still maintained high survival rate (Figure S11). Therefore, when the probe was not loaded with drug DOX, Ag_2_S@M-P-RGD exhibited a little toxicity to normal cells. For tumor cells, the viability of HeLa cells remained about 81.85±7.51 % after incubation with 200 μg/mL Ag_2_S@M-P-RGD for 24 h (Figure 4D), demonstrating high biocompatibility of probe. While the survival rate of MCF-7 cells was 91.17±4.01 %, probably because more targeting probe was uptake by HeLa cells. The targeting property of the probe also affected the cell survival. After incubation of HeLa cells with 200 μg/mL of Ag_2_S@M-P, cell survival rate of probe without RGD-modification (90.95±3.41 %) was higher than that of probe with RGD-modification, but there was no difference in cell survival rate for MCF-7 cells incubation with or without the targeting ligand of probe.

### *In vitro* antitumor effect

**Figure 5:**
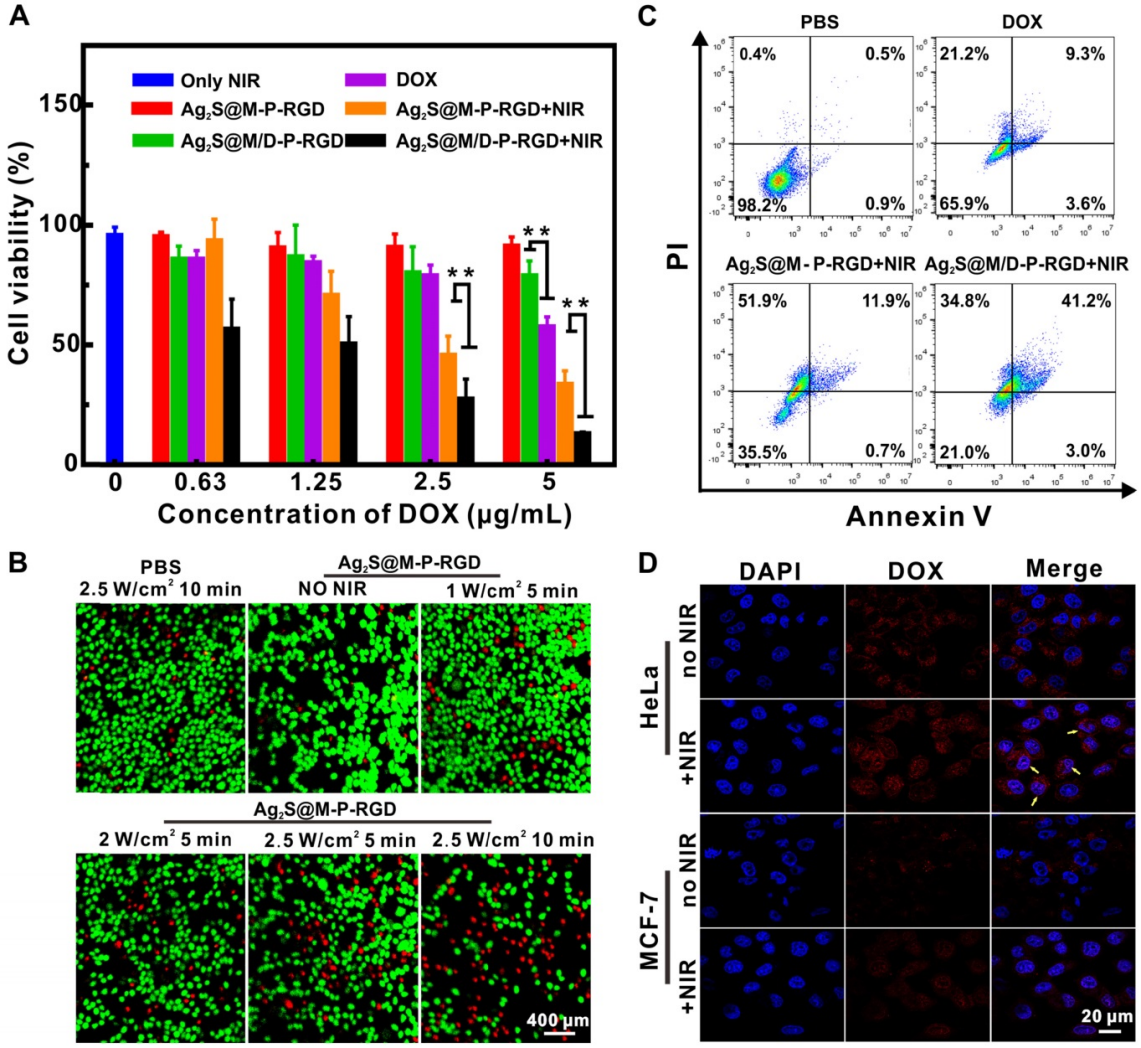
Evaluation of the therapy effect *in vitro*. MTT assay of survival rate of HeLa cells treated with different probes (A); calcein and PI stained fluorescence imaging images of HeLa cells were treated with Ag_2_S@M-P-RGD (100 µg/mL) irradiated with different intensity laser and time (B); flow cytometry of the apoptosis induced by different treatments (at the same concentration of DOX (5 µg/mL)) (C); CLSM images of HeLa and MCF-7 cells incubated with Ag_2_S@M/D-P-RGD irradiated with and without laser (D). **: *p*<0.01.

To further evaluate the chemo-photothermal synergistic therapeutic effect of Ag_2_S@M/D-P-RGD, the lethal effect of various treatment methods on tumor cells was studied (Figure 5A). It should be noted that since unit probe carried a certain amount of drug, DOX concentration here indicated probe concentration. The cell survival rate of Ag_2_S@M/D-P-RGD in absence of irradiation was 78.81±6.35 % even when the concentration was as high as 5 μg/mL, significantly higher than that of free DOX (*p*<0.01), illustrating that cys-P-RGD could effectively block DOX in the pore and prevent its leakage. When HeLa cells were treated by Ag_2_S@M-P-RGD (5 μg/mL), applying 10 min NIR irradiation (2.0 W/cm^-2^) could cause 66 % cell death, indicating that our probe killed most tumor cells through excellent PTT. To further study the photothermal effect of probe, cells were incubated with drug-free probe and treated with different laser intensities and time (Figure 5B). The results showed that the effect of only laser irradiation on the cell was negligible, whereas the cells after incubation with probe showed therapeutic effect even at lower intensity and cell death gradually increased with the rise of irradiation power and time. Since NIR laser irradiation was a focal light which could directly increase the temperature of probe and the resulting PTT could only kill cells irradiated by light, such local heat could not obviously influence the surrounding cell's viability. After HeLa cells of different treatment groups were double stained with Annexin V-FITC and PI, flow cytometry results showed that free DOX, Ag_2_S@M-P-RGD and Ag_2_S@M/D-P-RGD both irradiated with laser yielded approximately 34.1, 64.5 and 79.0 % apoptosis induction rate, respectively (Figure 5C). Local heating of light caused apoptosis in Ag_2_S@M-P-RGD group and apoptotic cells were mainly composed of necrotic cells losing cell membrane.

While drug-loaded Ag_2_S@M/D-P-RGD group induced apoptosis in approximate same proportion as necrotic cells, revealing significant effect of chemo-photothermal synergistic therapy. Drug-loaded probe combined with PTT could not only kill cells in the irradiated area, but also the surrounding cells. Similar results were obtained by MTT assay for cell therapy, when HeLa cells were incubated with low concentration of Ag_2_S@M/D-P-RGD (1.25 μg/mL), 10 min irradiation would cause about 50 % cell death. With the concentration reached 5 μg/mL, the cell survival rate was only 13.07±0.65 %, therapeutic effect was significantly higher than that of free DOX and PTT alone (*p*<0.01). As polypeptide could be used as blocking agent to realize on-demand controlled release of drug through thermosensitive characteristic, confocal images showed that DOX was mainly dispersed in cytoplasm and nucleus after drug-loaded probe was incubated with HeLa cells and exposed to laser (Figure 5D). However, the fluorescence signal of non-irradiated HeLa cells only existed in cytoplasm, indicating that DOX was still blocked in the pore without release. Together, the above results demonstrated that Ag_2_S@M/D-P-RGD targeted tumor cells with high expression of integrin α_v_β_3_, and NIR-controlled drug on/off release synergistic PTT had significant therapeutic effect.

### *In vivo* targeted NIR fluorescence-photoacoustic imaging and antitumor therapy

**Figure 6:**
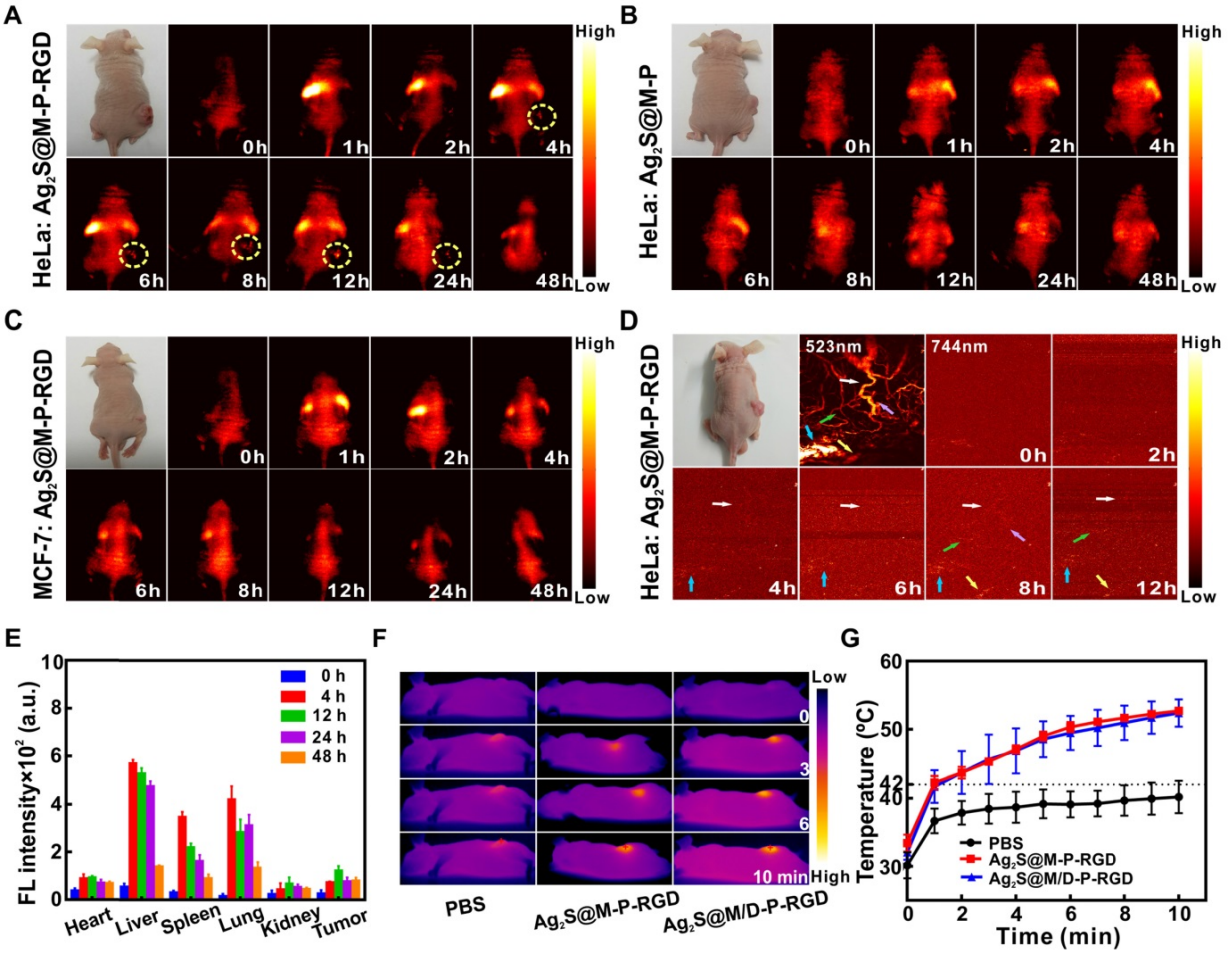
FLI at different time points after tail vein injection of Ag_2_S@M-P-RGD (100 mg/kg) into HeLa (A) and MCF-7 (C) tumor-bearing nude mice; FLI at different time points after tail vein injection of Ag_2_S@M-P (100 mg/kg) into HeLa tumor-bearing nude mice (B); PAI at different time points after tail vein injection of Ag_2_S@M-P-RGD (100 mg/kg) into HeLa tumor-bearing nude mice (D); distribution of Ag_2_S@M-P-RGD in major organs of HeLa tumor-bearing nude at different time points after tail vein injection (E); infrared thermal imaging (F) and temperature variation curves (G) of tumor site irradiated by laser after 12 h of intravenous injection 200 µL PBS, Ag_2_S@M-P-RGD (10 mg/mL) and Ag_2_S@M/D-P-RGD (10 mg/mL).

Multimodal imaging was used to detect the distribution of Ag_2_S@M/D-P-RGD in the tumor region for guiding therapy and monitoring therapeutic efficacy. After tail vein injection of Ag_2_S@M-P-RGD into HeLa tumor-bearing nude mice for 4 h, fluorescence signal at the tumor site began to enhance continuously, with the brightest fluorescence at 12 h and still observed at 48 h (Figure 6A). PAI was performed on the same tumor site of nude mice at different time points with 744 nm laser, tumor blood vessel began to appear after 4 h of injection and obvious vascular signal appeared at 8 h (Figure 6D). *In vitro* FLI of major organs and tumor showed the probe was firstly enriched in liver and lung, the strongest signal of tumor appeared 12 h after probe injection, then gradually metabolized with time (Figure 6E). To evaluate the targeting ability of probe *in vivo*, both tumor-bearing nude mice were intravenously injected with same concentration of Ag_2_S@M-P-RGD and Ag_2_S@M-P, respectively. Weak signal appeared at the tumor site of both gropes (Figure 6B and 6C) and the biodistributions were similar to that of the positive experimental group (Figure S12), however, the tumors showed weak fluorescence signal after some time due to passive targeting after injection of probes. Therefore, RGD-modified Ag_2_S@M-P-RGD exhibited the strongest signal and more persistent enrichment for HeLa tumor, indicating that our targeting probe was an excellent tracer probe owing to its strong fluorescence-photoacoustic signal and long residence time at tumor site. In addition, the potential of probe to induce hyperthermia effect *in vivo* was studied (Figure 6F and G), it was found tumor temperature only reached 38 °C in PBS group, indicating that the NIR laser was safe for normal tissue. In contrast, after 12 h of Ag_2_S@M-P-RGD injection, the temperature at the tumor site reached 53 °C under laser irradiation for 10 min. The DOX-loaded Ag_2_S@M/D-P-RGD still maintained similar photothermal conversion capability, inducing obvious cancer cell apoptosis.

How to ablate tumor without skin damage from laser irradiation remained an ongoing challenge for PTT. Researchers have proposed mild PTT that reduced the laser power to extend the exposure time or adopted multiple short-term laser exposure 46,47.

**Figure 7:**
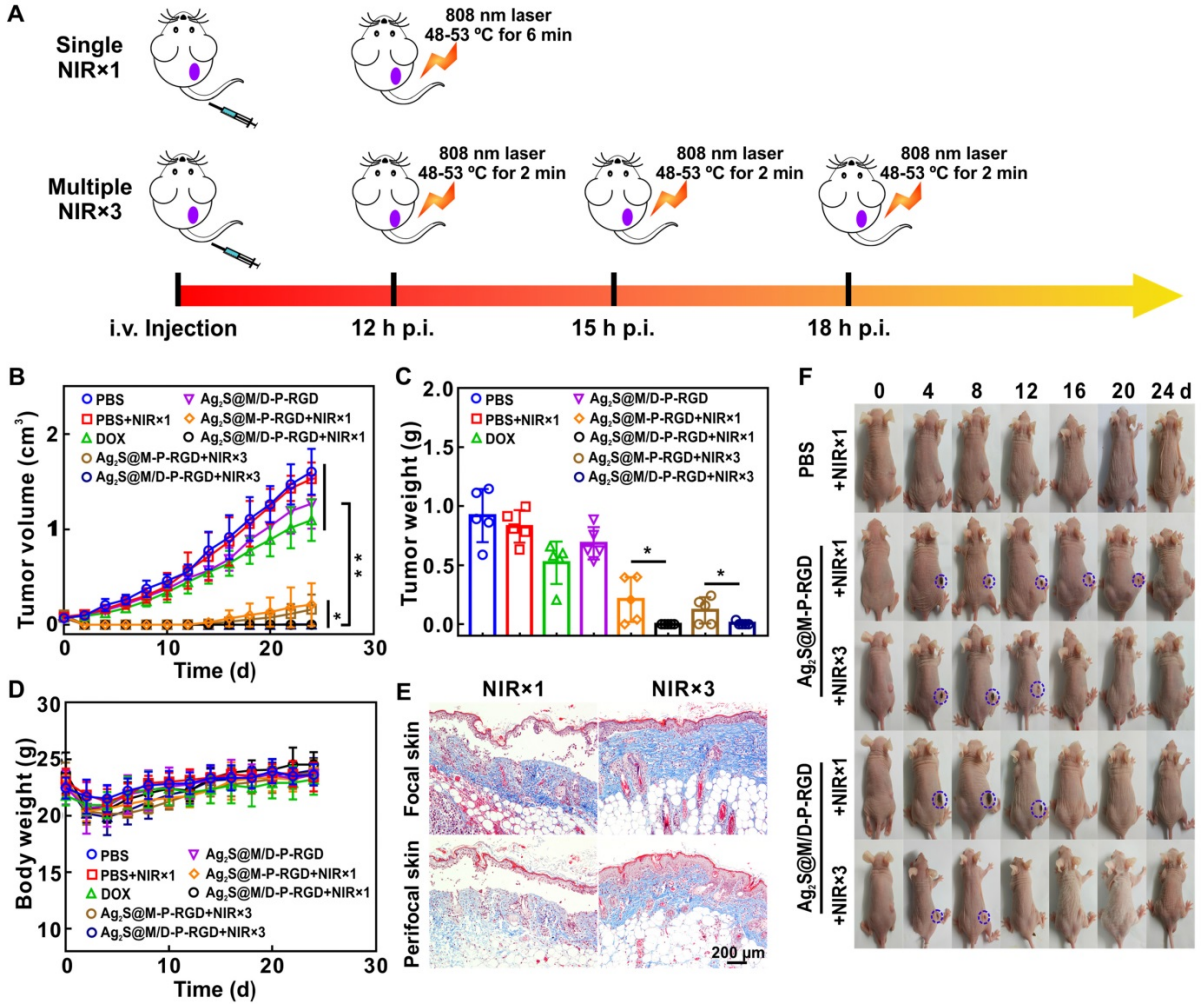
Schematic illustration of treatment route of single and multiple short-term laser irradiation (A); tumor growth after different treatments (B); body weights (D) and images (F) of mice with various treatments during 24 d; tumor mass in different groups at end of treatment (C); masson staining of tumor focal and peripheral skin of mice irradiated with single and multiple short-term laser irradiation (E). *: *p*<0.05, **: *p*<0.01.

Multiple short-term laser irradiation and controlled drug on/off release as required during *in vivo* therapy could reduce the skin damage caused by long-term laser irradiation and depress side effect caused by excessive drug release. To evaluate tumor inhibition effect of PTT with multiple short-term laser irradiation, the results of single and three times laser irradiation treatments were compared after intravenous injection of different probes into HeLa tumor-bearing nude mice. Single treatment groups applied 808 nm laser to irradiate tumor within the temperature range of 48~53 ºC for 6 min post-12 h of injection, while the tumor in three times treatment groups were irradiated for 2 min every 3 h in the same temperature range for three times to ensure the similar photothermal treatment conditions (Figure 7A). Analyzing the change in tumor volume (Figure 7B), the tumor growth rate in DOX group was slower than PBS group, but tumor volume was still increasing, indicating that chemotherapy alone was not enough to kill all tumor cells. However, the anti-tumor effect of DOX group was still better than that of drug-loaded Ag_2_S@M/D-P-RGD group without laser irradiation, due to the loaded DOX was still sealed in pore without leakage. After single or multiple laser exposure, treatment groups with or without drug loading showed excellent therapeutic effect at the initial stage of therapy. For the unloaded probe group, simple photothermal treatment was incomplete due to insufficient local heating, the tumor began to recur at 14 d after single laser irradiation treatment, and the similar phenomenon occurred at 16 d which dealt with three times irradiation. In comparison, the two chemo-photothermal synergistic therapy of drug-loaded groups rarely caused tumor relapse, and obtained the most efficient antitumor effect (*p*<0.05 or *p*<0.01). Similar to the above results, the effects of two chemo-photothermal synergistic therapy groups were significantly better than those of other groups (*p*<0.05 or *p*<0.01) after mice were sacrificed at 24 d and measured tumor masses (Figure 7C). In order to verify the therapeutic effects, tumors from mice after various treatments were collected for H&E and TUNEL staining (Figure S13). Compared with the non-irradiated group, the single and multiple laser-irradiated groups had a larger area of cell necrosis. Moreover, obviously enhanced apoptosis were observed in chemo-photothermal combined therapy of drug-loaded groups, and the results were consistent with changes in tumor volume. In addition, no noticeable body weight loss was observed in all groups during treatment process (Figure 7D), the blood biochemical analysis and the histological analysis of main organs for different groups also reveal that there were no pathological changes after treatment (Figure S14 and S15), indicating outstanding biocompatibility of probe and the treatment had no obvious toxicity to mice.

The above experiments showed that the synergistic treatment with multiple short-term laser irradiation could achieve the same tumor inhibition effect as the single long-term laser irradiation treatment, and tumor tissues all suffered scab formation and exfoliation after heating up (Figure 7F and Figure S16). However, the skin of mice exposed to multiple short-term laser irradiation showed smaller wound area and faster healing rate (8 d for NIR×3 group to healing while at least 12 d for NIR×1 group to recover). Masson staining was used to evaluate the collagen fiber of tumor skin and surrounding skin after different laser irradiation treatments (Figure 7E). The single long-time laser irradiation not only destroyed the epidermis, but also damaged the dermis and hair follicles. Whereas, the damage of multiple short-term treatment group was relatively small, the epidermis was slightly damaged, the dermis and hair follicles were basically intact. Furthermore, multiple mild hyperthermia effect could improve tumor oxygenation by enhancing blood flow into the tumor to further improve the efficacy of drug.

## Conclusions

In summary, a novel NIR laser-triggered multifunctional probe (Ag_2_S@M/D-P-RGD) with tumor targeted fluorescence-photoacoustic bimodal imaging and chemo-photothermal combined therapy was successfully designed and constructed. Based on the cys-P-RGD thermosensitive valve, DOX was blocked in dendritic mesoporous silica embedded Ag_2_S QD. The reversible polypeptide was manipulated by switching the laser on/off state by virtue of photothermal characteristic of Ag_2_S QD, which was capable of controlling the release amount of cargo through polypeptide structure change. Both* in vitro* and *in vivo* results demonstrated that Ag_2_S@M/D-P-RGD had satisfactory biocompatibility and targeting dual mode imaging ability, and mild photothermal therapy with multiple short-term laser irradiation could effectively inhibit tumor growth while reducing side effect of excessive drug release and skin damage. This novel theranostic nanocarrier integrated controllable chemo-photothermal synergistic therapy and real-time imaging might hold promise for efficient tumor therapy.

## Experimental Section

### Synthesis of dendritic mesoporous silica encapsulated Ag_2_S QD (Ag_2_S@M)

280 mg CTAB and 6 mg Ag_2_S QD were dissolved in 20 mL water and homogenized with ultrasound. The mixture was heated to 80 ºC and stirred rapidly to completely evaporate chloroform to obtain clear and transparent brown Ag_2_S/CTAB dispersion system. At the same time, 90 mg of TEA and 12 mg of NaSal (NaSal/CTAB molar ratio was 0.1: 1) were dissolved in 5 mL water and preheated at 80 ºC for 30 min before adding to the above system. After the mixed system was equilibrated for 1 h, 1.5 mL TEOS was added dropwise and gently stirred for 2 h. The resulting Ag_2_S@M was purified by repeated centrifugation (10000 rpm, 10 min) to remove the unreacted reagent by water and ethanol, then re-dispersed in 50 mL ethanol containing ammonium nitrate (10 mg/mL) and heated to 75 ºC for 6 h to remove CTAB template. After the same purification methods mentioned above, it was resuspended in 30 mL ethanol and reacted with 150 µL MPTES at 60 ºC for 20 h to modify sulfydryl, then washed as above. Ag^+^ concentration was measured by graphite furnace atomic absorption method.

### DOX loading and polypeptide blocking pore

20 mg Ag_2_S@M and 2 mg DOX were dispersed into 5 mL water. After stirring for 24 h, 2 mL cys-P-RGD (3 mg/mL) was added and reacted with BMOE coupling agent for 2 h to cap the pore. The mixture was repeated centrifugation (12000 rpm, 10 min) with PBS (0.1 M, pH 7.4) to clean free DOX to obtain DOX-loaded nanoparticle (Ag_2_S@M/D-P-RGD); cys-P was also used to prepare control probe without targeting RGD (Ag_2_S@M/D-P). BSA had a free cysteine that could be bonded to mercapto-modified Ag_2_S@M surface *via* BMOE and was used as control to an analyze the active role of cys-P-RGD. 10 mg Ag_2_S@M/D-P-RGD was added in 50 µL hydrofluoric acid to dissolve silica, and pH was adjusted to neutrality to determine DOX amount by analyzing fluorescence intensity at 590 nm, loading content (%) = (DOX weight in probe)/(probe weight)×100 % and encapsulation efficiency (%) = (DOX weight in probe)/(DOX initial weight)×100 %.

### *In vitro* antitumor therapy

To investigate the photothermal property of the probe *in vitro*, HeLa cells were seeded in glass-bottom Petri dishes and Ag_2_S@M-P-RGD (100 µg/mL) was added after overnight cultured. After further culture for 4 h, PBS was used to remove the unbound probe. Then each well was added 200 µL PBS and irradiated at different laser intensities (0.5~2.5 W/cm^2^) and different treatment time. The cells were stained with calcein and propidine iodide (PI) to observe the effect of photothermal therapy.

The combined antitumor therapy was studied based on MTT assay. After the HeLa cells were seeded in the 96-well plate for overnight culture, various probes with different concentrations (0.63, 1.25, 2.5 and 5 µg/mL DOX) were added for incubation for 4 h. After washing with PBS to remove unbound probe, cells were irradiated with 808 nm laser (2.0 W/cm^2^) for 10 min or without any treatment. Then cells were grown for another 24 h and measured cell viability.

The extent of apoptosis *in vitro* was determined by Annexin V-FITC/propidium iodide (PI) double staining apoptosis assay. HeLa cells were seeded in 6-well plate and cultured overnight. The cells were treated with free DOX, Ag_2_S@M-P-RGD and Ag_2_S@M/D-P-RGD at the same concentration of DOX (5 µg/mL) for 4 h. After cleaning, they were exposed to laser (2.0 W/cm^2^) for 10 min and continuously cultured for 24 h. Subsequently, cells were stained with Annexin V-FITC and PI according to the protocol and analyzed by FC500 Flow cytometer (Beckman Coulter, USA).

### *In vivo* combined antitumor efficacy and safety evaluation

HeLa tumor-bearing nude mice with an average volume of ~200 mm^3^ were randomly divided into eight groups (n=5) and intravenously injected with 100 mg/kg of different probes: (I) PBS, (II) PBS + NIR×1,(III) free DOX, (IV) Ag_2_S@M/D-P-RGD, (V) Ag_2_S@M-P-RGD + NIR×1, (VI) Ag_2_S@M/D-P-RGD + NIR×1, (VII) Ag_2_S@M-P-RGD + NIR×3, (VIII) Ag_2_S@M/D-P-RGD + NIR×3. NIR×1 referred to irradiation of tumor site with 808 nm laser (2.0 W/cm^2^) for 6 min at 48-53 ºC after injection of 12 h; NIR×3 meant that the tumor was heated up to 48-53 ºC for 2 min three times by the same laser irradiation after 12 h, 15 h and 18 h after injection. The treated HeLa tumor-bearing nude mouse of each group was sacrificed after laser irradiation and tumors were fixed with 4 % paraformaldehyde, dehydrated, embedded and sectioned for H&E and TUNEL staining. The tumor volume and body weight of mice were measured for 24 d. When the experiments were finished, mice were sacrificed and major organs (heart, liver, spleen, lungs, kidneys and small intestine) were collected and weighed. The contents of ALT, aspartate aminotransferase (AST), blood urea nitrogen (BUN) and creatinine (CREA) in blood were analyzed, and organs were stained with hematoxylin/eosin (H&E). Visceral index was calculated as follows, organ mass/mice mass×100 %. All animal experiments were approved by Animal Experimental Ethics Committee of Huazhong University of Science and Technology.

## Abbreviations

PTT: photothermal therapy
db: desthiobiotin
DOX: doxorubicin
QD_s_: quantum dots
Ag(DDTC): Diethyldithiocarbamic acid silver salt
CTAB: hexadecyltrimethylammonium bromide
TEOS: tetraethylorthosilicate
MPTES: (3-mercaptopropyl) trimethoxysilane
PDI: polydispersity index
TUNEL: terminal deoxynucleotidyl transferase dUTP nick end labeling
