# Supplementary material for: A near-infrared light-controlled smart nanocarrier with reversible polypeptide-engineered valve for targeted fluorescence-photoacoustic bimodal imaging-guided chemo-photothermal therapy
Source: Theranostics. 2019 Oct 14;9(25):7666–79. doi: 10.7150/thno.37047 (PMC6831479; doi:10.7150/thno.37047)
Supplement: Supplementary file 1 — Supplementary figures and tables. [file thnov09p7666s1.pdf]

Supporting Information

A near-infrared light-controlled smart nanocarrier with reversible polypeptide-engineered valve for targeted fluorescence-photoacoustic bimodal imaging-guided chemo-photothermal therapy

Cheng Li [a,b], Xiao-Quan Yang [a], Jie An [a], Kai Cheng [a], Xiao-Lin Hou [a], Xiao-Shuai Zhang [a], Xian-Lin Song [a], Kai-Chen Huang [d], Wei Chen [a], Bo Liu [a], Yuan-Di Zhao [a,c,]*, Tian-Cai Liu [d,e]*

[a] Britton Chance Center for Biomedical Photonics at Wuhan National Laboratory for Optoelectronics-Hubei Bioinformatics & Molecular Imaging Key Laboratory, Department of Biomedical Engineering, College of Life Science and Technology, Huazhong University of Science and Technology, Wuhan 430074, Hubei, P. R. China

[b] Beijing Advanced Innovation Center for Big Data-Based Precision Medicine, School of medicine, Beihang University, Beijing 100191, P. R. China

[c] Key Laboratory of Biomedical Photonics (HUST), Ministry of Education, Huazhong University of Science and Technology, Wuhan 430074, Hubei, P. R. China

[d] Institute of Antibody Engineering, School of Laboratory Medicine and Biotechnology, Southern Medical University, Guangzhou 510515, Guangdong, P. R. China

[e] Guangdong Provincial Key Laboratory of Construction and Detection in Tissue Engineering, Southern Medical University, Guangzhou 510515, Guangdong, P. R. China

* Corresponding author. Tel/Fax: +86-27-8779-2202. Email address: zydi@mail.hust.edu.cn (Y.D. Zhao); liutc@smu.edu.cn (T.C. Liu).

Experimental Section

Materials

Diethyldithiocarbamic acid silver salt (Ag(DDTC), 98 %) was purchased from Aladdin Industrial Co., Ltd. (Shanghai, China); octadecene (ODE, 90 %), hexadecyltrimethylammonium bromide (CTAB), tetraethylorthosilicate (TEOS), 3-aminopropyltriethoxysilane (APTES), (3-mercaptopropyl) trimethoxysilane (MPTES), bis-maleimidoethane (BMOE), β-mercaptoethanol, isopropyl-β-D-thiogalactoside (IPTG), ampicillin and kanamycin were purchased from Sigma-Aldrich (St. Louis, MO, USA); doxorubicin hydrochloride (DOX) was purchased from Sangon Biotech Industrial Co., Ltd. (Shanghai, China); dodecylmercaptan (DT, 98%), triethanolamine (TEA), sodium salicylate (NaSal) and dimethyl sulfoxide (DMSO) were purchased from Sinopharm Chemical Reagent Co., Ltd. (Shanghai, China). Ni-NTA separation column was purchased from Qiagen Co., Ltd. (Shanghai, China). All chemicals and reagents were used as received without any further purification.

Characterizations

Probe morphology characterization and element analysis were performed by Tecnai G2 20 U-Twin (FEI, U.S.), HT7700 (Hitachi, Japan) transmission electron microscopy and EDX spectroscopy. The absorption spectra were measured by UV-2550 UV-vis spectrophotometer (Shimadzu, Japan). The fluorescence spectra of DOX were obtained by LS-55 fluorescence spectrophotometer (PerkinElmer, USA) and near infrared fluorescence (NIR) spectra were determined by NIRQUEST512-1.7 fiber spectrometer (Ocean Optics, USA). ZS90 ZetaSizer (Malvern, UK) was employed to characterize the size and surface potential of probe, the specific surface area (BET) and pore size distribution (BJH) of nanoparticle were analyzed by ASAP 2020 fully automatic surface area analyzer (Micromeritics Instrument, USA). MDL-III-808-2.5W laser (Changchun New Industries, China) was used for laser irradiation, and thermal imaging was recorded by EasIR-9 infrared thermal imager (Wuhan Guide, China). NIR imaging system[1] and photoacoustic imaging system[2] were built up by our laboratory.

Synthesis of hydrophobic Ag_2S QD

The method was modified according to the previous literature [3], the steps were as follows, 76.8 mg Ag(DDTC), 30 g ODE and 6 g DT were heated to 90 ℃ under Ar protection and maintained for 10 min to remove the water in the system. The reaction was further heated to 150 ℃ and quenched by *n*-hexane after maintaining the temperature for 10 min. After the solution was cooled to room temperature, centrifuged (12000 rpm, 10 min) and washed twice with acetone. The hydrophobic Ag_2S QD was re-dispersed in chloroform for later use.

Synthesis and purication of the polypeptide

Cys-P and cys-P-RGD plasmids were constructed by conventional genetic engineering method. The polypeptide was prepared and purified according to previous methods in our laboratory [4,5]. PQE9Pcys and PQE9RGDPcys plasmids were transfected into *E. Coli* strain M15, respectively. Bacterial was cultured at 37 ℃ in LB liquid medium supplemented with ampicillin (50 mg/L) and kanamycin (25 mg/L). When the OD_{600} value of the bacterial solution reached 0.7~1.0, 1 mM IPTG was added to induce the expression of target polypeptide. After 4 h of continuous culture, bacteria were harvested by centrifugation (12,000 rpm, 30 min) and lysed in 8 M urea (pH 8.0). The bacteria were lysed for 3 times through repeated freeze-thaw at -80 ℃ and centrifuged at 12,000 rpm for 10 min to isolate the crude polypeptide extract. The 6 ×histidine tag encoded in the pQE9 vector allowed purification of the polypeptide on the Ni-NTA resin by affinity chromatography in accordance with the denaturation protocol proposed by Qiagen. After dialysis, the products were obtained by vacuum freeze drying, cys-P without targeting ability (MS: 7050.3 Da, the theoretical calculation of molecular weight: 7053.8 Da) and cys-P-RGD with targeting ability (MS: 8483.5 Da, the theoretical calculation of molecular weight: 8487.2 Da).

Photothermal effect and photothermal conversion efficiency

300 μL Ag_2S@M at different concentrations (0~8 mg/mL) was irradiated with 808 nm laser (2 W/cm^2) for 5 min at a distance of 5 cm, and same concentration (4 mg/mL) Ag_2S@M was irradiated at different laser intensities (0.5~2.5 W/cm^2). The temperature changes were recorded by thermal

imager. In photothermal stability test, 300 μL Ag_2S@M was irradiated with laser (2 W/cm^2) for 10 min and then naturally cooled to room temperature (n=6). The photothermal conversion efficiency (η) was determined by following equation [6], $\eta=\frac{hS(T_{\max}-T_{\text{surr}})-Q_{\text{dis}}}{I\ (1-10^{-A_{808}})}$, I was irradiation power to be 800 mW, A_{808} was absorption of probe at 808 nm (A_{808}=0.884), $T_{\max}$-$T_{\min}$ was 27.5 ℃, τ_s and hS were calculated to be 154.9 s and 8.134; same method measured water (300 μL) as a control and Q_{Dis} was calculated to be 26.48. On the basis of data, η could be obtained.

Protein absorption

BSA was used as a model protein to evaluate protein adsorption on nanoparticle in different environments according to previous study [7]. Ag_2S@M and Ag_2S@M-P-RGD nanoparticles were incubated with BSA at 37 ℃ in PBS (0.01M) at pH 7.4 and 5.4, respectively. The final concentrations of nanoparticles and BSA were 0.4 and 0.3 mg/mL, respectively. After 12 and 24 h, solution was centrifuged to collect supernatant, the BSA concentration was calculated by BSA assay kit (BCA protein concentration assay kit, Beyotime) according to the protocol. The absorbed BSA was calculated using the following equation: $q=(C_i-C_f)V/m$, C_i and C_f were the initial and final BSA concentrations before and after BSA absorption; V was the total solution volume; m is the weight of particles added into solution.

Immunological analysis

In order to investigate the effect of reducing immune response after polypeptide modification of nanoparticles, the inflammatory cytokines (IL-6, IL-1β and TNF-α) were analyzed. Ag_2S@M and Ag_2S@M-P-RGD (100 μg/mL) were incubated with macrophage RAW 264.7 at 37 ℃ for 12 h. The mRNA expression levels were quantified by real-time reverse transcription polymerase chain reaction (qRT-PCR). Fluorescent dye was AceQ qPCR SYBR Green Master Mix (High ROX Premixed) (Vazyme Biotech Co., Ltd) and the primer pairs were as follows: IL-6, 5'-CTG CAA GAG ACT TCC ATC CAG-3' (forward) and 5'-AGT GGT ATA GAC AGG TCT GTT GG-3' (reverse); IL-1β, 5'-GAA ATG CCA CCT TTT GAC AGT G-3' (forward) and 5'-TGG ATG CTC TCA TCA GGA CAG-3' (reverse); TNF-α, 5'-CCG CGA CGT GGA ACT GG-3' (forward) and 5'-GGC CAT TTG

GGA ACT TCT CAT-3' (reverse); GAPDH, 5'-TGA CCT CAA CTA CAT GGT CTA CA-3' (forward) and 5'-CTT CCC ATT CTC GGC CTT G-3' (reverse).

Hemolysis Assay

Blood compatibility was evaluated by hemolysis assay. Fresh mice blood was extracted from orbital vein and stabilized with heparin. 2 mL whole blood was diluted with PBS to 4 mL and centrifuged at 3500 rpm for 5 min to isolate red blood cell (RBCs). The RBCs were further washed and finally diluted to 20 mL PBS. Different concentrations of Ag_2S@M and Ag_2S@M-P-RGD were incubated with RBCs at 37 ℃ for 4 h, water as positive control and PBS as negative control. The absorbance of the supernatants from each group was measured using ELX808IU microplate reader (Biotek, USA) at 570 nm. The hemolysis percentage was calculated as follows, hemolysis percentage = $(OD_{test}-OD_{negative\ control})/(OD_{positive\ control}-OD_{negative\ control})\times100$ %.

Circular dichroism of cys-P-RGD

Polypeptide cys-P-RGD with concentration of 100 μM was scanned by J-810 circular dichroism spectrometer (Jasco, Japan) with temperature gradient in range of 20~70 ℃. The wavelength spectra were measured over a range from 190 to 300 nm with a step size of 1 nm.

Cell culture

HeLa and MCF-7 cell lines were cultured in DMEM culture medium (Gbico, Invitrogen) containing 10 % FBS and incubated in humidified atmosphere containing 5 % CO_2 at 37 ℃. In the experiment, 0.25 % trypsin was used for digestion.

NIR-triggered drug release

Two groups of 5 mg drug-loaded Ag_2S@M/D-P-RGD were dispersed in 2 mL PBS (0.01 M, pH 7.4) and incubated in 37 ℃ water bath and one group was irradiated with laser (2 W/cm^2, 10 min) at different time points. Both groups were centrifuged (12000 rpm, 10 min) to obtain supernatant and DOX contents were measured according to the standard curve.

Endocytosis and cytotoxicity

HeLa and MCF-7 cells were seeded in 6-well plate (5×10^4 cells per well) and grown for 24 h. Replaced serum-free medium, 100 μg/mL Ag_2S@M-P-RGD and Ag_2S@M-P were cultured with cells for another 4 h. After terminating the culture, cells were washed with PBS to remove free probe, then immobilized with 4 % paraformaldehyde and collected for fluorescence and photoacoustic imaging.

For confocal imaging of cellular uptake of probe, HeLa and MCF-7 cells were seeded in glass-bottom Petri dishes and incubated for 24 h. The cells were treated with Ag_2S@M/D-P-RGD and Ag_2S@M/D-P (C_{DOX} = 1 μg/mL) for another 4 h and washed with PBS. After irradiation of laser (2 W/cm^2) for 10 min or not, different treatment groups were imaged by FV1000 confocal microscopy (Olympus, Japan). The RGD blocking group was pre-incubated with RGD (10 μg/mL) for 2 h before probe addition. DAPI was nuclear dye and LysoTracker® Green DND was lysosomal dye.

In order to evaluate the *in vitro* safety of probe, MTT assay was used to analysis cell viability of HeLa and MCF-7 cells. Cells were seeded in 96-well plate (1×10^4 cells per well) and incubated overnight. Subsequently, cells were treated with different amounts of Ag_2S@M-P-RGD andAg_2S @M-P (25, 50, 100 and 200 μg/mL) for 24 h. After 20 μL MTT (5 mg/mL) was added into each well to incubate for another 4 h, the medium was discarded and 150 μL DMSO was added to solubilize formazan crystal, ELX808IU microplate reader (Biotek, USA) was used to measure the absorption at 490 nm. Untreated cells were used as negative control, n=4.

In vivo **FLI-PAI and thermal imaging**

4-5 weeks Balb/C nude mice (male, SPF grade) were injected subcutaneously with 100 μL resuspended HeLa cells (1×10^6) or MCF-7 cells (2×10^6) PBS to induce tumor formation. The tumor began to be treated when its volume reached ~100 mm^3 ($0.5\times$length$\times$width^2). For *in vivo* FLI-PAI, Ag_2S@M-P-RGD or Ag_2S@M-P (100 mg/kg) were administered by tail vein injection in tumor-bearing mice and acquired signals at different time points (0, 1, 2, 4, 6, 8, 12, 24 and 48 h). For thermal imaging, HeLa tumor-bearing nude mice were intravenously injected with 200 μL PBS, Ag_2S@M-

P-RGD (10 mg/mL) or Ag_2S@M/D-P-RGD (10 mg/mL), 808 nm laser irradiated the tumor site for 10 min after 24 h of incubation and the temperature was recorded by thermal imager.

Pharmacokinetics and distribution of Ag_2S@M-P-RGD

Ag_2S@M and Ag_2S@M-P-RGD (100 mg/kg) was injected *i.v.* into female Kunming mice. After 5 min, 1, 3, 6, 12 and 24 h after injection, blood was collected from the mice and dissolved in nitric acid to obtain the total amount of Ag^+ by graphite furnace atomic absorption spectrometry. HeLa tumor-bearing nude mice were injected with Ag_2S@M-P-RGD (100 mg/kg) intravenously, and the fluorescence intensity of different organs was measured by FL imaging system at different time points.

Statistical analysis

All data were presented as mean±SD unless otherwise stated. All the experiments were performed at least in triplicate. The statistical significance was determined using two-tailed Student's test (*$p<0.05$, **$p<0.01$) unless otherwise stated.

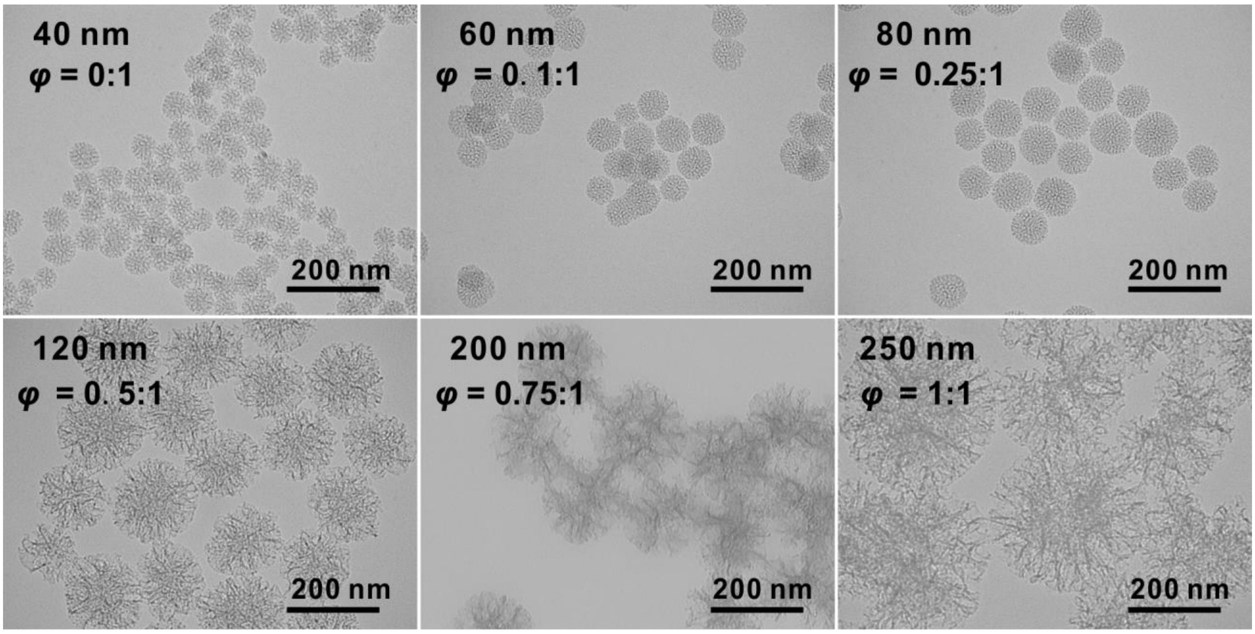

Figure S1. TEM images of the effect of different NaSal/CTAB molar ratio (φ) on size and structure of Ag_2S@M.

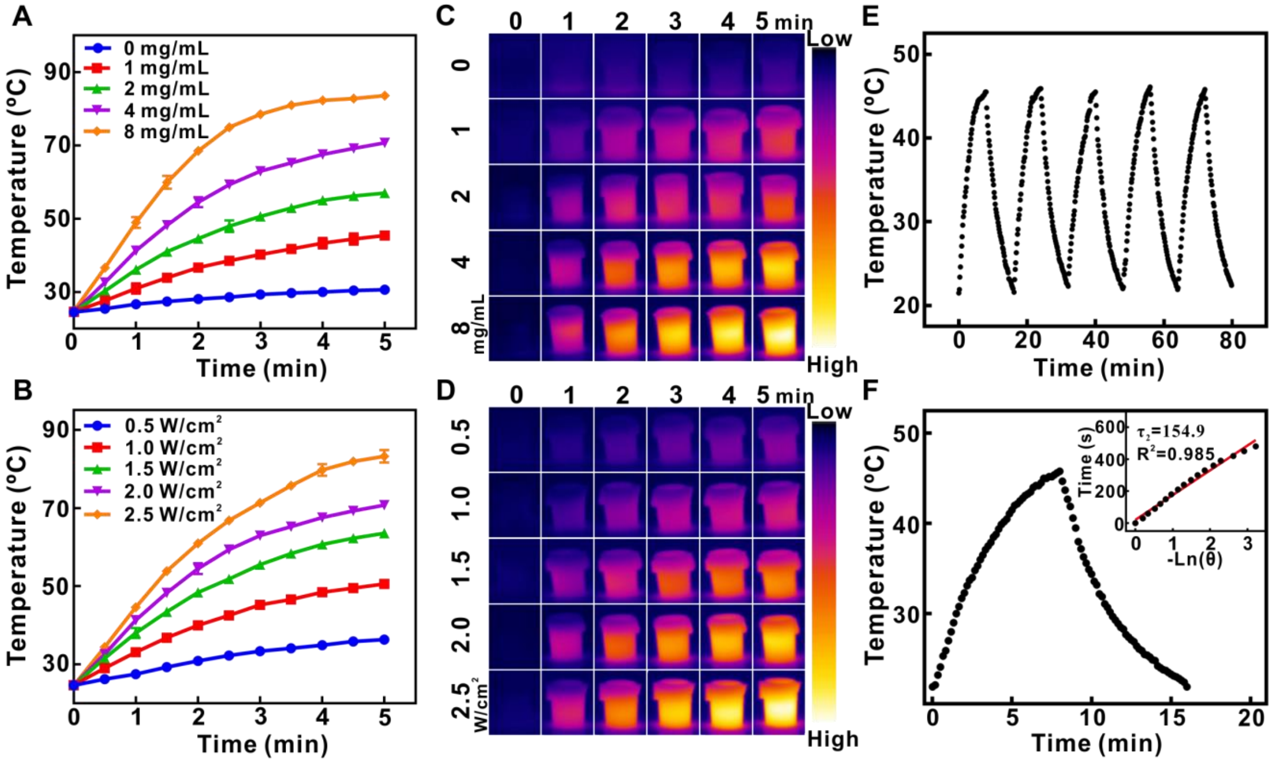

Figure S2. The photothermal heating curves of Ag_2S@M (A) and infrared thermal images (C) of Ag_2S@M at different concentrations under same laser irradiation (2.0 W/cm^2); photothermal heating curves (B) and infrared thermal images (D) of Ag_2S@M (4.0 mg/mL) under various power intensities; heating of Ag_2S@M in water for five laser on/off cycles with 808 nm laser (2.0 W/cm^2) (E); heating and cooling curve of Ag_2S@M under laser irradiation (F).

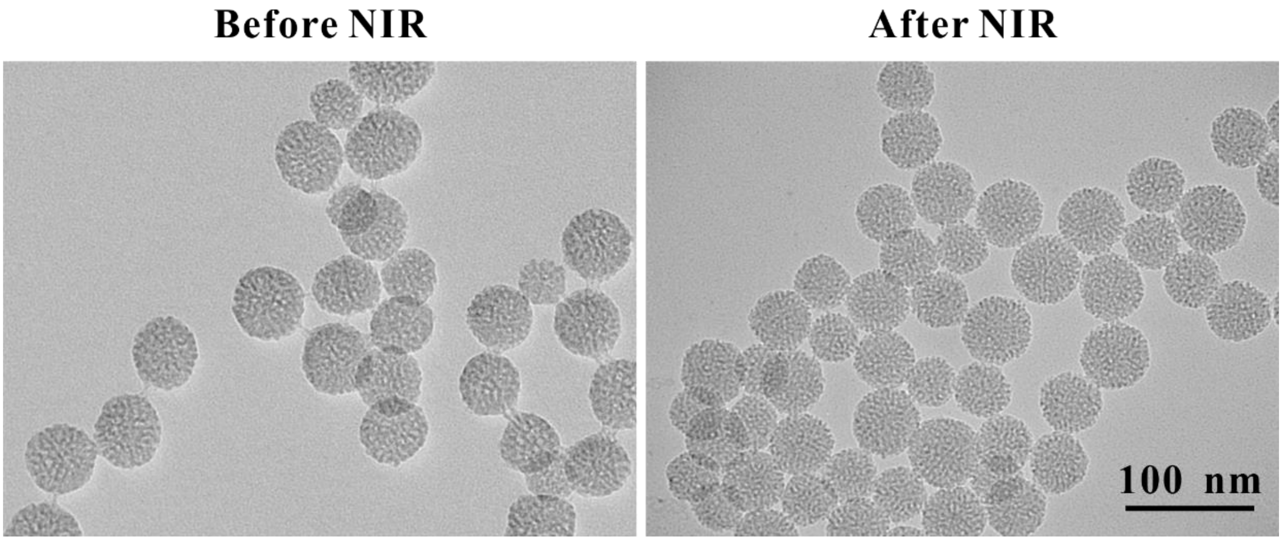

Figure S3. TEM images of Ag_2S@M before and after laser irradiation.

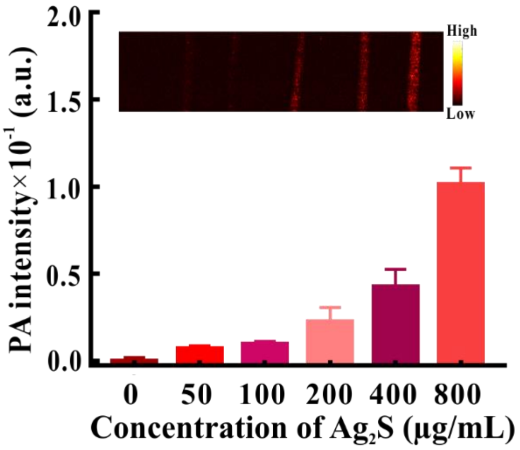

Figure S4. Photoacoustic images of Ag_2S at different concentrations.

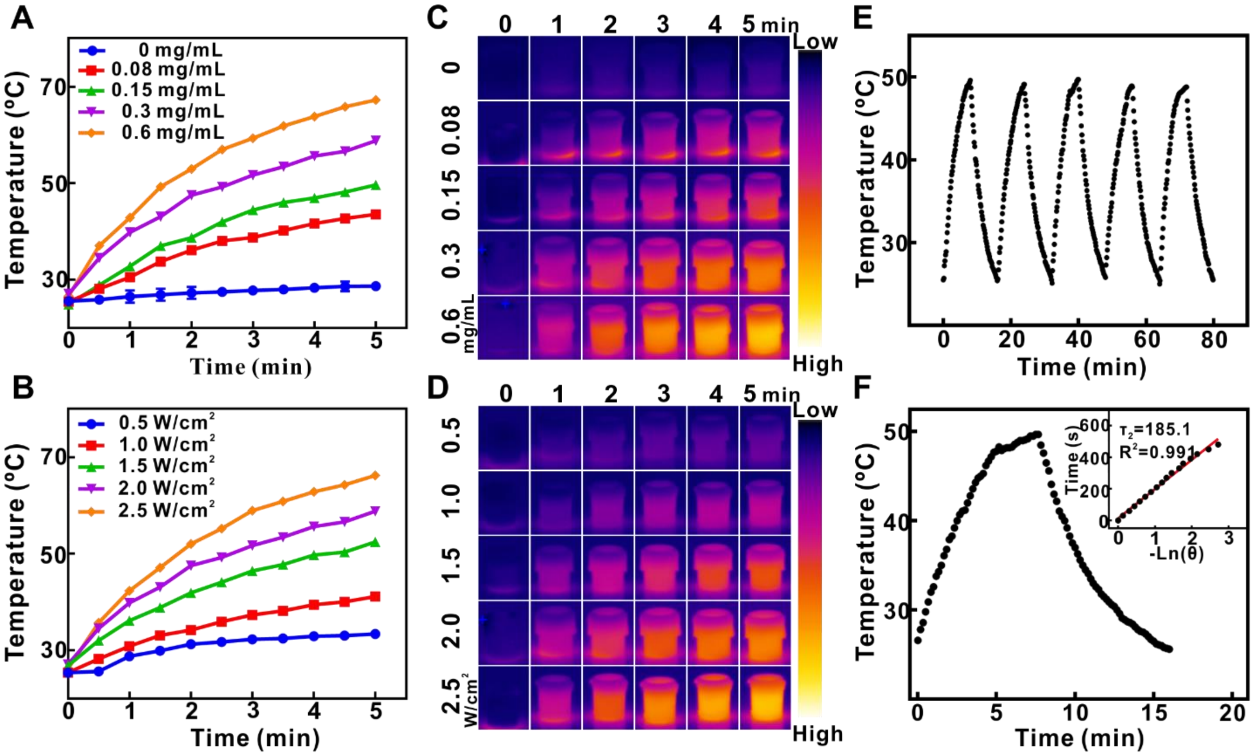

Figure S5. The photothermal heating curves of Ag_2S (A) and infrared thermal images (C) of Ag_2S at different concentrations under same laser irradiation (2.0 W/cm^2); photothermal heating curves (B) and infrared thermal images (D) of Ag_2S (0.3 mg/mL) under various power intensities; heating of Ag_2S in water for five laser on/off cycles with 808 nm laser (2.0 W/cm^2) (E); heating and cooling curve of Ag_2S under laser irradiation (F).

In order to compare Ag_2S-loaded MSN with the same amount of Ag_2S nanoparticles, the oil-soluble Ag_2S QD was encapsulated by using the hydrophobic structure of Pluronic F-127 to obtain hydrophilic Ag_2S nanomicelle. The photoacoustic imaging and photothermal effect of Ag_2S@P (η=29.49%) were evaluated by using the same amount of Ag_2S (Ag^+ concentration was measured by graphite furnace atomic absorption).

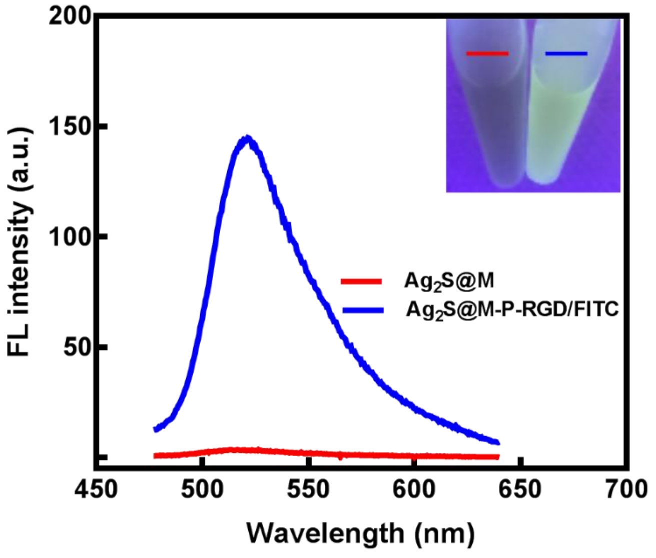

Figure S6. Fluorescence spectra and image (inset)of Ag_2S@M and Ag_2S@M-P-RGD/FITC.

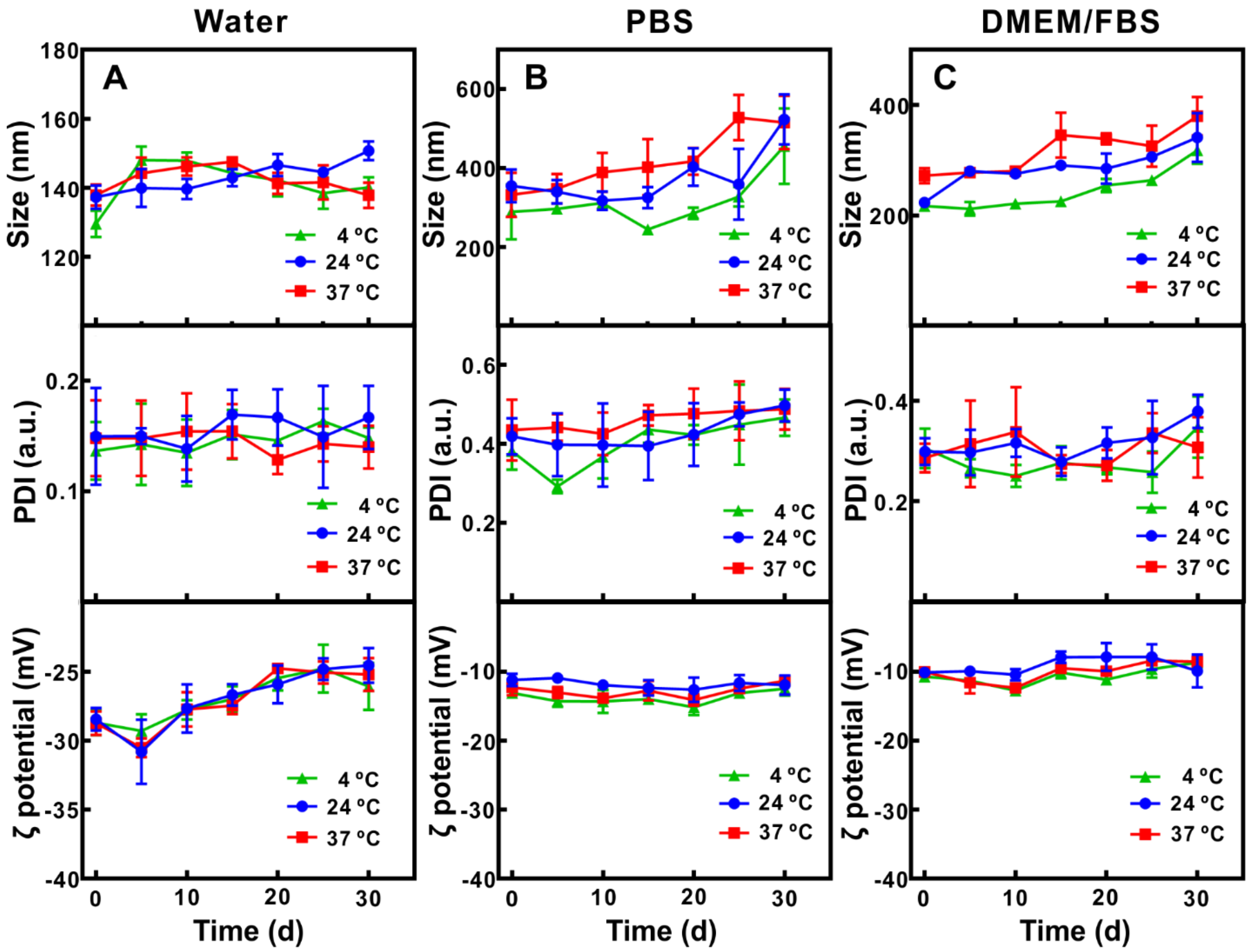

Figure S7. The change of hydrated particle size, PDI and zeta potential of Ag_2S@M-P-RGD stored in water (A), PBS (B) and DMEM medium with 10% FBS (C) at 4, 24 and 37 ℃, respectively.

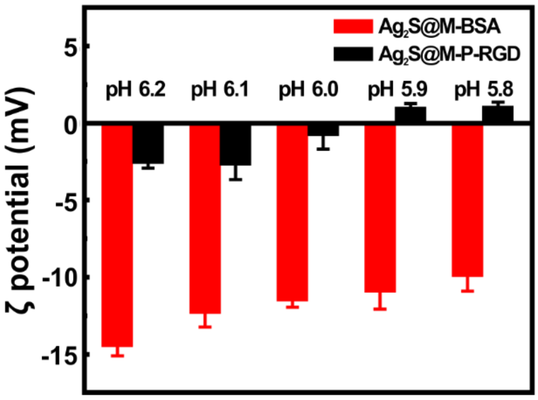

Figure S8. Zeta potential changes of Ag_2S@M-BSA and Ag_2S@M-P-RGD under different pH conditions.

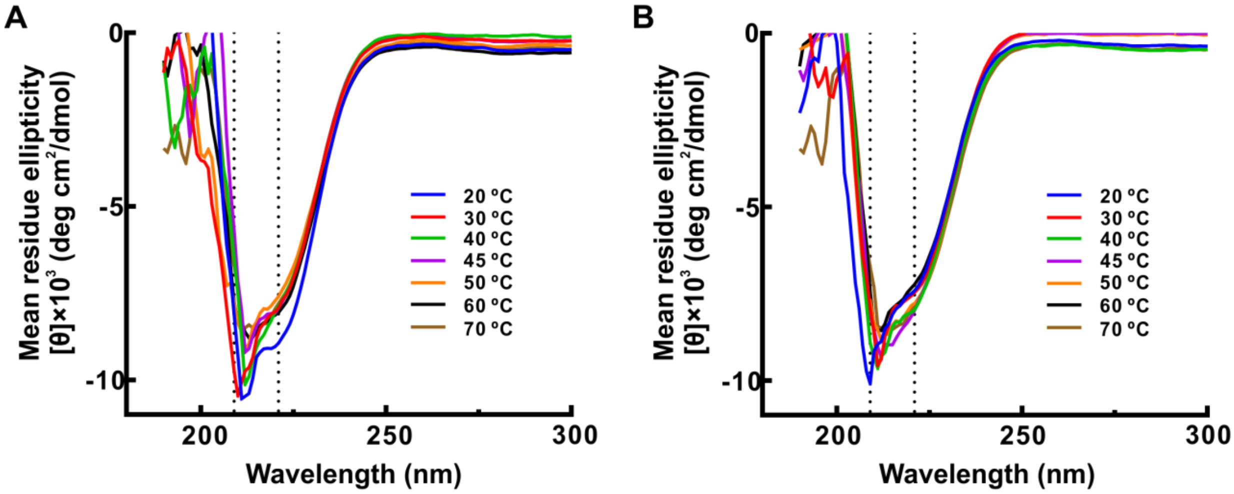

Figure S9. CD spectra of polypeptide cys-P-RGD temperature gradient heating (A) and cooling (B).

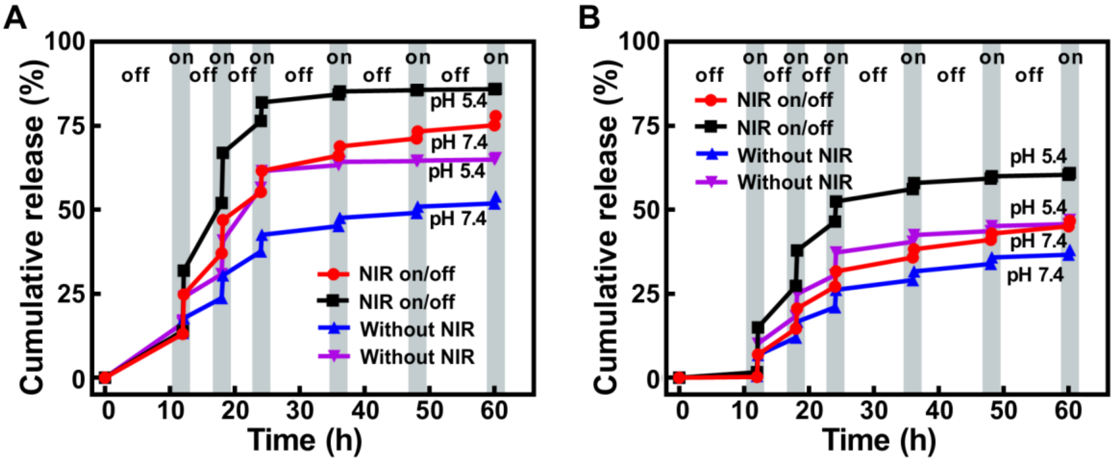

Figure S10. DOX release curves of Ag_2S@M/D (A) and Ag_2S@M/D-BSA (B) with and without laser irradiation at pH 7.4 and 5.4.

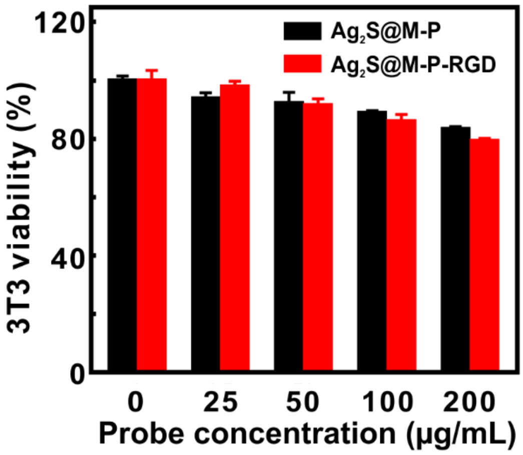

Figure S11. Survival rate of 3T3 cells incubated with Ag_2S@M-P and Ag_2S@M-P-RGD at different concentrations.

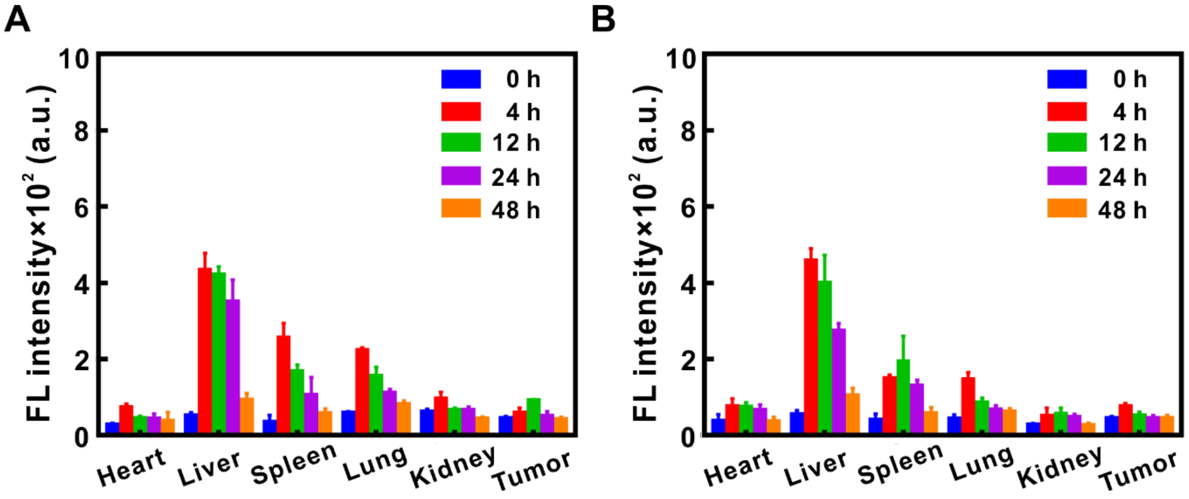

Figure S12. Distribution of Ag_2S@M-P in major organs of HeLa tumor-bearing nude at different time points after tail vein injection (A); distribution of Ag_2S@M-P-RGD in major organs of MCF-7 tumor-bearing nude mice at different time points after tail vein injection (B).

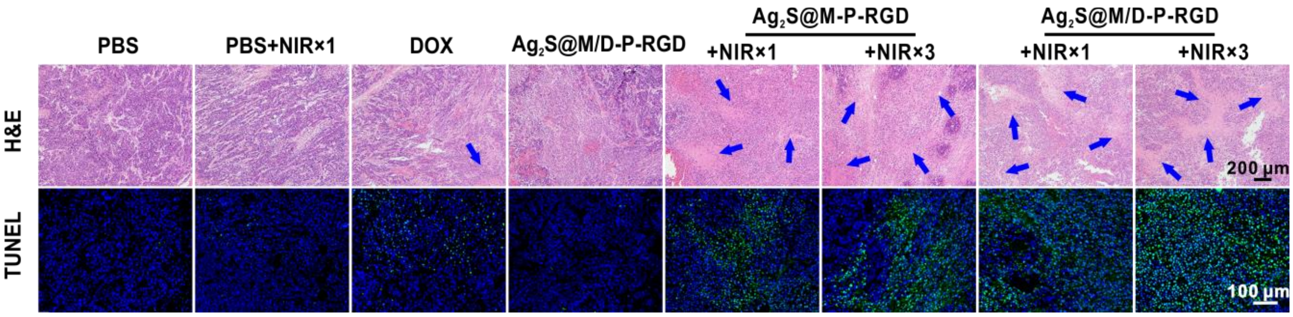

Figure S13. H&E and TUNEL staining of tumors in each treatment group.

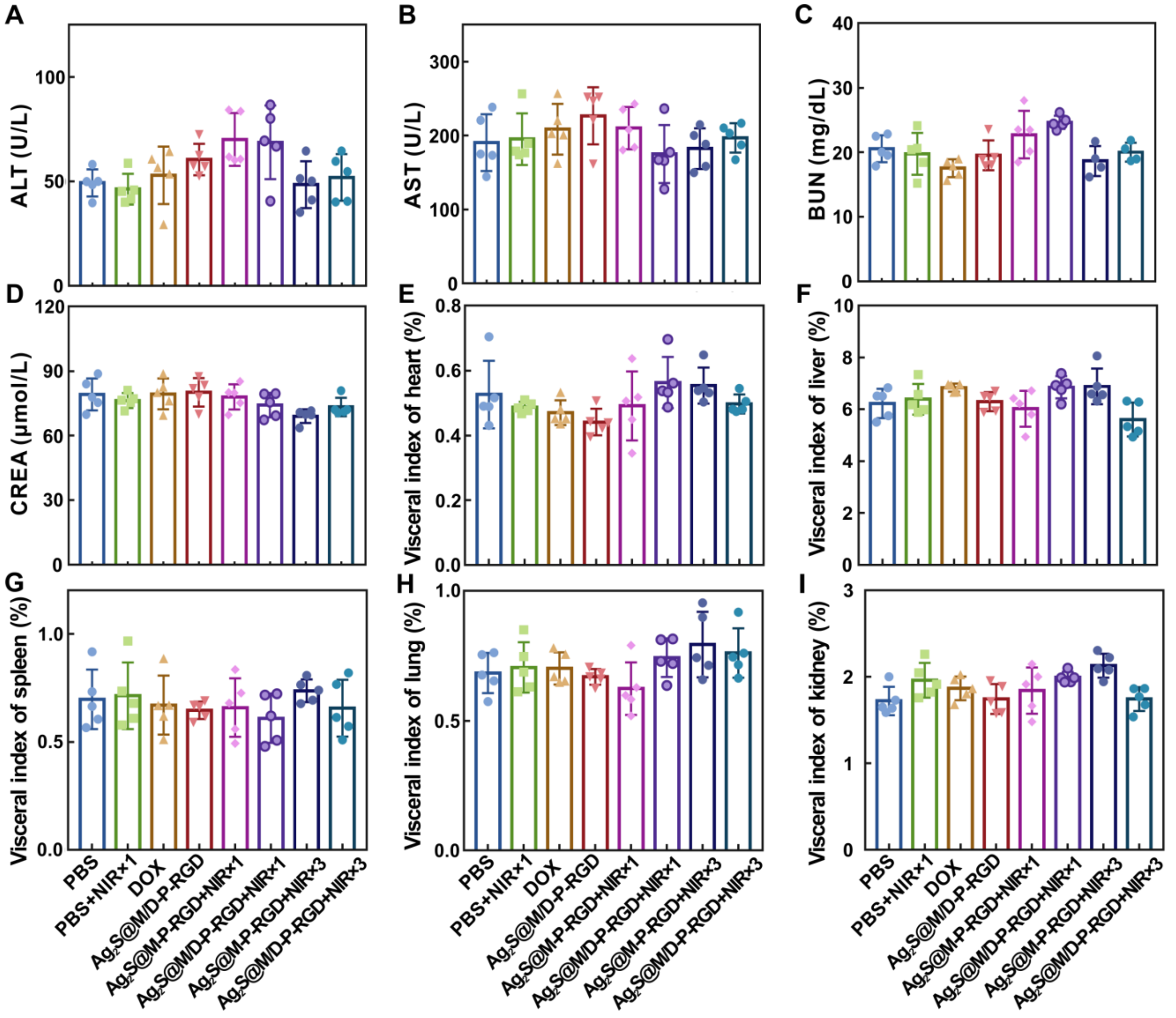

Figure S14. Evaluation of blood compatibility and biological safety. Blood biochemical analysis (A~D) and visceral index of major organs (E~I) at the end of different treatment groups.

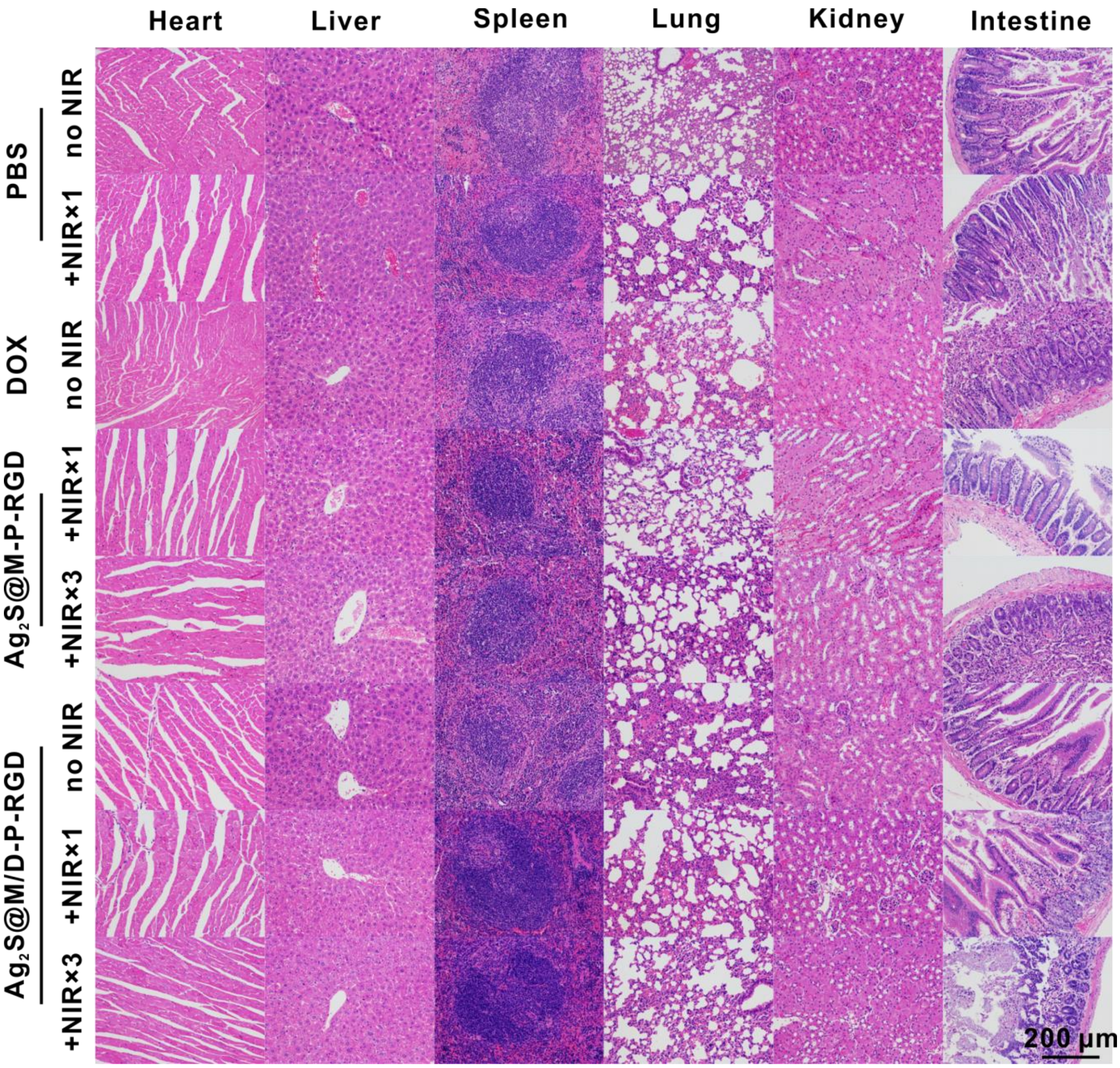

Figure S15. H&E staining of major organs at the end of different treatment groups.

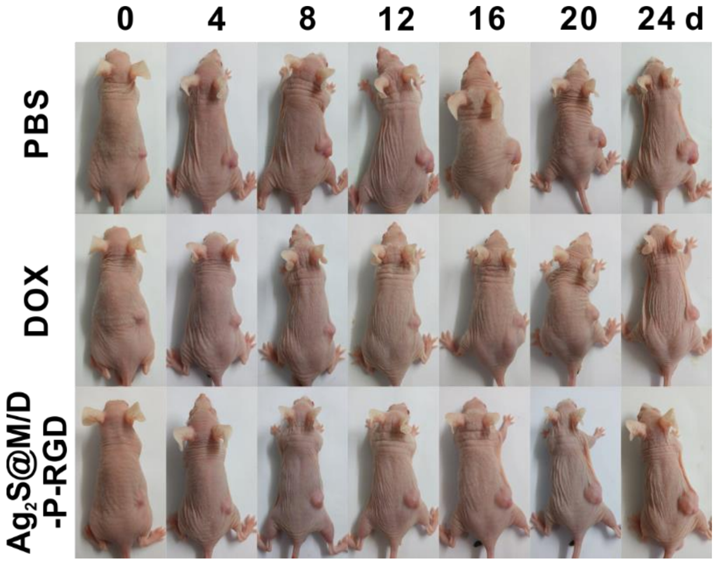

Figure S16. Images of mice with various treatments during 24 d.

Reference

[1] Wang K, Wang Q, Luo Q, Yang X. Fluorescence molecular tomography in the second near-infrared window. Opt Express. 2015; 23: 12669-79.

[2] Liu Y, Yang X, Gong H, Jiang B, Wang H, Xu G, et al. Assessing the effects of norepinephrine on single cerebral microvessels using optical-resolution photoacoustic microscope. J Biomed Opt. 2013; 18: 076007.

[3] Hong G, Robinson JT, Zhang Y, Diao S, Antaris AL, Wang Q, et al. In vivo fluorescence imaging with Ag_2S quantum dots in the second near-infrared region. Angew Chem Int Ed Engl. 2012; 51: 9818-21.

[4] Yao MH, Yang J, Du MS, Song JT, Yu Y, Chen W, et al. Polypeptide-engineered physical hydrogels designed from the coiled-coil region of cartilage oligomeric matrix protein for three-dimensional cell culture. J Mater Chem B. 2014; 2: 3123-32.

[5] Yao MH, Yang J, Song JT, Zhao DH, Du MS, Zhao YD, et al. Directed self-assembly of polypeptide-engineered physical microgels for building porous cell-laden hydrogels. Chem Commun. 2014; 50: 9405-8.

[6] Roper DK, Ahn W, Hoepfner M. Microscale heat transfer transduced by surface plasmon resonant gold nanoparticles. J Phys Chem C. 2007; 111: 3636-41.

[7] Zhang M, Xu C, Wen L, Han MK, Xiao B, Zhou J, et al. A hyaluronidase responsive nanoparticle-based drug delivery system for targeting colon cancer cells. Cancer Res. 2016; 1681.
